# Supplementary material for: Long-term impacts of an urban sanitation intervention on enteric pathogens in children in Maputo city, Mozambique: study protocol for a cross-sectional follow-up to the Maputo Sanitation (MapSan) trial 5 years postintervention
Source: BMJ Open. 2023 Jun 8;13(6):e067941. doi: 10.1136/bmjopen-2022-067941 (PMC10254709; doi:10.1136/bmjopen-2022-067941)
Supplement: Author's manuscript [file bmjopen-2022-067941.draft_revisions.pdf]

BMJ Open

BMJ Open is committed to open peer review. As part of this commitment we make the peer review history of every article we publish publicly available.

When an article is published we post the peer reviewers' comments and the authors' responses online. We also post the versions of the paper that were used during peer review. These are the versions that the peer review comments apply to.

The versions of the paper that follow are the versions that were submitted during the peer review process. They are not the versions of record or the final published versions. They should not be cited or distributed as the published version of this manuscript.

BMJ Open is an open access journal and the full, final, typeset and author-corrected version of record of the manuscript is available on our site with no access controls, subscription charges or pay-per-view fees (http://bmjopen.bmj.com).

If you have any questions on BMJ Open's open peer review process please email info.bmjopen@bmj.com

BMJ Open

Long-term impacts of an urban sanitation intervention on enteric pathogens in children in Maputo city, Mozambique: study protocol for a cross-sectional follow-up to the Maputo Sanitation (MapSan) trial five years post-intervention

Journal:	*BMJ Open*
Manuscript ID	bmjopen-2022-067941
Article Type:	Protocol
Date Submitted by the Author:	31-Aug-2022

Complete List of Authors:	Holcomb, David; The University of North Carolina at Chapel Hill Gillings School of Global Public Health, Environmental Sciences and Engineering<br>Monteiro, Vanessa; Centro de Investigação e Treino em Saúde da Polana Caniço, Instituto Nacional de Saúde<br>Capone, Drew; Indiana University, Department of Environmental and Occupational Health, School of Public Health<br>António, Virgílio; Centro de Investigação e Treino em Saúde da Polana Caniço, Instituto Nacional de Saúde<br>Chiluvane, Márcia; Centro de Investigação e Treino em Saúde da Polana Caniço, Instituto Nacional de Saúde<br>Cumbane, Victória; Centro de Investigação e Treino em Saúde da Polana Caniço, Instituto Nacional de Saúde<br>Ismael, Nália; Division of Biotechnology and Genetics, Instituto Nacional de Saúde<br>Knee, Jackie ; London School of Hygiene and Tropical Medicine Faculty of Infectious and Tropical Diseases<br>Kowalsky, Erin; The University of North Carolina at Chapel Hill Gillings School of Global Public Health, Department of Environmental Sciences and Engineering<br>Lai, Amanda; University of North Carolina at Chapel Hill Gillings School of Global Public Health, Environmental Science and Engineering<br>Linden, Yarrow; The University of North Carolina at Chapel Hill Gillings School of Global Public Health, Department of Environmental Sciences and Engineering<br>Mataveia, Elly; Centro de Investigação e Treino em Saúde da Polana Caniço, Instituto Nacional de Saúde<br>Nala, Rassul; Instituto Nacional de Saúde<br>Rao, Gouthami; The University of North Carolina at Chapel Hill Gillings School of Global Public Health, Department of Environmental Sciences and Engineering<br>Ribeiro, Jorge; Centro de Investigação e Treino em Saúde da Polana Caniço, Instituto Nacional de Saúde<br>Cumming, Oliver; London School of Hygiene and Tropical Medicine, Department of Disease Control<br>Viegas, Edna; Centro de Investigação e Treino em Saúde da Polana Caniço, Instituto Nacional de Saúde<br>Brown, Joe; University of North Carolina at Chapel Hill Gillings School of Global Public Health, Environmental Science and Engineering

Keywords:	Gastrointestinal infections < GASTROENTEROLOGY, Epidemiology < INFECTIOUS DISEASES, PUBLIC HEALTH

SCHOLARONE™
Manuscripts

TITLE

Long-term impacts of an urban sanitation intervention on enteric pathogens in children in Maputo city, Mozambique: study protocol for a cross-sectional follow-up to the Maputo Sanitation (MapSan) trial five years post-intervention

AUTHORS

David Holcomb[1,2], Vanessa Monteiro[3], Drew Capone[1,4], Virgílio António[5], Márcia Chiluvane,[3] Victória Cumbane[3], Nália Ismael[5], Jackie Knee[6], Erin Kowalsky[1], Amanda Lai[1], Yarrow Linden[1], Elly Mataveia[3], Rassul Nalá[7], Gouthami Rao[1], Jorge Ribeiro[3], Oliver Cumming[6], Edna Viegas[3], Joe Brown[1,*]

AFFILIATIONS

1. Department of Environmental Sciences and Engineering, Gillings School of Global Public Health, University of North Carolina at Chapel Hill, Chapel Hill, North Carolina, USA

2. Department of Epidemiology, Gillings School of Global Public Health, University of North Carolina at Chapel Hill, Chapel Hill, North Carolina, USA

3. Centro de Investigação e Treino em Saúde da Polana Caniço, Instituto Nacional de Saúde, Maputo, Mozambique

4. Department of Environmental and Occupational Health, School of Public Health, Indiana University, Bloomington, Indiana, USA

5. Division of Biotechnology and Genetics, Instituto Nacional de Saúde, Marracuene, Mozambique

For peer review only - http://bmjopen.bmj.com/site/about/guidelines.xhtml

6. Department of Disease Control, Faculty of Infectious and Tropical Diseases, London School of Hygiene and Tropical Medicine, London, UK

7. Division of Parasitology, Instituto Nacional de Saúde, Marracuene, Mozambique

CORRESPONDING AUTHOR

* Joe Brown; 135 Dauer Drive, Chapel Hill, NC, USA, 27599-7435; T: (919) 966-7892; E: joebrown@unc.edu

For peer review only

ABSTRACT

Introduction

We previously assessed the effect of an onsite sanitation intervention in informal neighborhoods of urban Maputo, Mozambique on enteric pathogen detection in children after two years of follow-up (Maputo Sanitation [MapSan] trial, clinicaltrials.gov: NCT02362932). We found significant reductions in *Shigella* and *Trichuris* prevalence but only among children born after the intervention was delivered. In this study, we assess the health impacts of the sanitation intervention after five years among children born into study households post-intervention.

Methods and analysis

We are conducting a cross-sectional household study of enteric pathogen detection in child stool and the environment in the 16 MapSan study neighborhoods at compounds (household clusters sharing sanitation and outdoor living space) that received the pour-flush toilet and septic tank intervention at least five years prior or meet the original criteria for trial control sites. We are enrolling at least 400 children (ages 29 days-60 months) in each treatment arm. Our primary outcome is the prevalence of 22 bacterial, protozoan, and soil transmitted helminth (STH) enteric pathogens in child stool using the pooled odds ratio across the outcome set to assess the intervention effect. Secondary outcomes include the individual pathogen detection prevalence and gene copy density of 27 enteric pathogens (including viruses); mean height-for-age (HAZ), weight-for-age (WAZ), and weight-for-height (WHZ) z-scores; prevalence of stunting, underweight, and wasting; and the 7-day period-prevalence of caregiver-reported diarrhea. All analyses are adjusted for pre-specified covariates and examined for effect measure modification by age. Environmental samples from study households and the public domain are assessed for

For peer review only - http://bmjopen.bmj.com/site/about/guidelines.xhtml

pathogens and fecal indicators to explore environmental exposures and monitor disease transmission.

Ethics and dissemination

Study protocols have been reviewed and approved by human subjects review boards at the Ministry of Health, Republic of Mozambique and the University of North Carolina at Chapel Hill. De-identified study data will be deposited at https://osf.io/e7pvk/.

Trial registration number

ISRCTN86084138

For peer review only

For peer review only - http://bmjopen.bmj.com/site/about/guidelines.xhtml

KEYWORDS

Enteric infection; diarrheal disease; molecular pathogen detection; environmental microbiology; wastewater-based epidemiology

STRENGTHS AND LIMITATIONS OF THIS STUDY

- Follow-up to the first controlled health impact trial of an urban on-site sanitation intervention after at least 5 years of intervention conditions. Previous studies have focused on WASH intervention impacts up to 2-3 years after delivery, which may be insufficient time to realize impacts.
- Primary study endpoint is molecular detection of multiple enteric pathogens in child stool, which unambiguously indicates previous exposure to specific sanitation-related pathogens; enteric pathogens are also assessed across multiple environmental compartments to explore environmental exposures and monitor disease transmission.
- Primary outcome is the overall impact of the intervention on enteric pathogen exposure using a novel pooled estimate of the treatment effect across a pre-specified set of enteric pathogens.
- As an observational evaluation of an existing intervention, sample size is constrained by the number of eligible children residing at study sites and selection bias arising from differential enteric pathogen-related mortality may be present, particularly among older age groups.
- As a cross-sectional study, there is potential for confounding bias in our estimates of the intervention impacts on child health; in particular, the presence of the intervention itself may have influenced the desirability of the intervention sites and thus the socioeconomic status of their residents, which may be associated with increased pathogen exposures.

For peer review only - http://bmjopen.bmj.com/site/about/guidelines.xhtml

INTRODUCTION

There is a high burden of childhood enteric infections associated with poor environmental conditions, with multiple enteric pathogens frequently detected in stool within the first year of life [1–3]. This is also the period with the highest incidence of diarrhea, which remains a leading cause of child mortality in low- and middle-income countries (LMIC) and has long been associated with stunted growth [4–6]. However, many diarrheal episodes are not attributable to infectious etiologies [3,7], while the etiology of attributable diarrhea varies widely by setting and only certain diarrheal pathogens are consistently implicated in reduced linear growth [2,8,9]. Far more prevalent is asymptomatic enteric pathogen shedding in stool [3,7,10–14], which may be more strongly associated with poor growth than diarrheal illness [2,15–17], potentially by contributing to intestinal inflammation, gut permeability, and nutrient malabsorption in a condition known as environmental enteric dysfunction (EED) [18–21]. In addition to the diverse negative impacts of stunting [22], specific adverse health outcomes associated with enteric infection and EED include delayed cognitive development [23–25] and reduced oral vaccine efficacy [26,27].

Water, sanitation, and hygiene (WASH) interventions aim to prevent diarrhea and improve child growth by interrupting fecal-oral pathogen transmission [28]. However, recent rigorous WASH intervention trials have demonstrated inconsistent and often limited impacts on child health [29–35]. Methodological limitations of such distal outcome measures notwithstanding [36,37], these findings are consistent with the interventions insufficiently interrupting environmental transmission and exposure to enteric pathogens [38]. Combined WASH interventions with high fidelity and adherence reduced stool-based detection of *Giardia*, hookworm, and possibly

For peer review only - http://bmjopen.bmj.com/site/about/guidelines.xhtml

Ascaris among 30 month-old children (but not younger children) and enteric viruses in rural Bangladesh [10,39,40], *Ascaris* in rural Kenya [41], and the number of co-detected parasites in rural Zimbabwe [11], but not bacterial pathogens or those most associated with stunting in any setting [2]. The sanitation-only intervention arm in rural Bangladesh reduced *Trichuris* prevalence in child stool [39] and may have reduced pathogenic *Escherichia coli* on child hands in more crowded households [42], while a sanitation intervention in rural Cambodia did not impact any pathogens measured in child stool [35]. Viewed collectively, a clear picture emerges of pervasive childhood polymicrobial exposures that were not meaningfully prevented by low-cost WASH interventions [43–45].

The preceding trials were all conducted in rural settings, but rapid urbanization has led to exceptional growth of densely populated informal settlements that lack basic services and present unique health challenges [46,47]. We have previously investigated whether an onsite sanitation intervention delivered in low-income neighborhoods of urban Maputo, Mozambique reduced enteric pathogen prevalence in child stool [48]. Although we similarly found no evidence of an effect on combined prevalence of pre-specified enteric pathogens in our primary analyses [34], additional evidence suggests that the sanitation intervention may have reduced exposures to enteric pathogens in the environment. The intervention was delivered with high fidelity and was widely used by intervention households after two years [49]. Exploratory sub-group analyses indicated that 24 months after the intervention, *Shigella* prevalence was halved (-51%; 95% confidence interval [CI]: -15%, -72%) and *Trichuris* prevalence reduced by three-quarters (-76%; 95% CI: -40%, -90%) among children born into the study compounds after the intervention was implemented, relative to children born into the control compounds after baseline [34].

For peer review only - http://bmjopen.bmj.com/site/about/guidelines.xhtml

Furthermore, overall pathogen prevalence, pathogen counts, *E. coli* gene copy density, and the individual prevalence of *Ascaris* and pathogenic *E. coli* were all significantly reduced in soil at the intervention latrine entrance [50,51], suggesting the intervention effectively contained human excreta. While animals have been implicated as major sources of pathogen exposure in other settings [52], only companion animals were frequently present at study households and a locally validated indicator of poultry fecal contamination (the most commonly observed non-companion animal type) was rarely detected in household environments [53]. Conversely, indicators of human fecal contamination were widespread [51] and the two pathogens most impacted by the intervention—*Shigella* and *Trichuris*—are considered anthroponotic [54], suggesting that human excreta was a primary source of enteric pathogens in our dense, urban setting and that human-associated pathogens were impacted by sustained exposure to the intervention.

Enteric pathogen carriage is highly age-dependent [8,12], but we were previously limited by the timing of our follow-up survey (24 months post-intervention) to children under two years of age when assessing impacts on those born into the intervention. Furthermore, it has been suggested that the typically short (1-2 year) follow-up periods often used in WASH evaluations may be inadequate for any longer-term benefits to manifest, potentially contributing to the limited observed effects [43]. Accordingly, we are conducting a cross-sectional follow-up study to better understand the long-term impacts of the sanitation intervention five years after it was implemented on child enteric pathogen exposures and shedding, diarrheal disease, and growth among children born into study households post-intervention.

For peer review only - http://bmjopen.bmj.com/site/about/guidelines.xhtml

STUDY OBJECTIVES

Primary objective

To measure the long-term effect of a shared, onsite urban sanitation intervention on the pooled prevalence of pre-specified enteric pathogen targets detected in children's stool at least 60 months post-intervention.

Hypotheses

H1. The risk of stool-based enteric pathogen detection among children 29 days-60 months old is reduced for children born into households that previously received the sanitation intervention.

H2. Children born into households that previously received the sanitation intervention experience delayed exposure to enteric pathogens relative to comparably aged children from non-intervention households, reflected in a greater reduction in the risk of enteric pathogen detection among younger age groups and attenuated reduction in risk among older children.

Secondary objectives

1. To measure the effect of a shared, onsite urban sanitation intervention program on the individual prevalence and density of pre-specified enteric pathogen targets detected in children's stool and environmental samples at least 60 months post-intervention.
2. To measure the effect of a shared, onsite urban sanitation intervention program on child growth and the prevalence of caregiver-reported diarrhea in children at least 60 months post intervention.

For peer review only - http://bmjopen.bmj.com/site/about/guidelines.xhtml

METHODS AND ANALYSIS

Study setting

This household-based study is being conducted in 11 *bairros* (neighborhoods) in the *Nhlamankulu* District and 5 *bairros* in the *KaMaxaquene* District of Maputo city, Mozambique. Households in these densely populated, low-income neighborhoods typically cluster into self-defined compounds delineated by a wall or boundary that share outdoor living space and sanitation facilities. Study activities with participating households are conducted primarily in the compound shared outdoor space by staff from the *Centro de Investigação e Treino em Saúde da Polana Caniço* (Polana Caniço Health Research and Training Center; CISPOC) in Maputo, Mozambique, from which the study is implemented and managed.

Study design

Urban sanitation intervention

The nongovernmental organization (NGO) Water and Sanitation for the Urban Poor (WSUP) implemented a sanitation intervention in 2015-2016 at approximately 300 compounds in informal urban neighborhoods of Maputo under a larger program led by the Water and Sanitation Programme (WSP) of the World Bank [48]. In these compounds, ranging between three and twenty-five households each, existing unhygienic shared latrines were replaced with pour-flush toilets and a septic tank with a soak-away pit for the liquid effluent. Two intervention designs employing the same sanitation technology were implemented, with approximately 50 communal sanitation blocks (CSBs) and 250 shared latrines (SLs) constructed across 11 neighborhoods that exhibited diversity across density and other key characteristics (susceptibility to flooding,

For peer review only - http://bmjopen.bmj.com/site/about/guidelines.xhtml

relative poverty, access to water and sanitation infrastructure) [12,53,55]. SLs served compounds with fewer than 21 residents and provided a single cabin with the intervention toilet, while CSBs provided an additional cabin for every 20 compound residents and other amenities including rainwater harvesting, municipal water connections and storage, and washing, bathing and laundry facilities [34,56].

Original controlled before-and-after study

To evaluate the impact of the WSUP sanitation intervention on child health, we conducted the Maputo Sanitation (MapSan) trial, a controlled before-and-after study of enteric pathogen detection in child stool (clinicaltrials.gov: NCT02362932) [48]. WSUP selected intervention compounds using the following criteria: (1) residents shared sanitation in poor condition as determined by an engineer; (2) the compound was located in the pre-defined implementation neighborhoods; (3) there were no fewer than 12 residents; (4) residents were willing to contribute financially to construction costs; (5) sufficient space was available for construction of the new facility; (6) the compound was accessible for transportation of construction materials and tank-emptying activities; (7) the compound had access to a legal piped water supply; and (8) the groundwater level was deep enough for construction of a septic tank. Control compounds were selected according to criteria 1, 3, 4, and 7 from the 11 neighborhoods where the intervention was implemented and 5 additional neighborhoods with comparable characteristics [12].

The MapSan trial recruited an open cohort at three time-points: baseline (pre-intervention), 12 months post-intervention, and 24 months post-intervention; children were eligible to participate if older than 1 month at the time of enrollment and under 48 months at baseline. We enrolled

For peer review only - http://bmjopen.bmj.com/site/about/guidelines.xhtml

intervention and control compounds concurrently to limit any differential effects of seasonality or other secular trends on the outcomes. We found no evidence that the sanitation intervention reduced the combined prevalence of 12 bacterial and protozoan enteric pathogens (the pre-specified primary outcome), the individual prevalence of any single bacterial, protozoan, STH, or viral pathogen, or the period prevalence of caregiver-reported diarrhea 12 and 24 months post-intervention [34]. However, exploratory analyses indicated the intervention may have been protective against bacterial and soil-transmitted helminth infections among the cohort who were born into the intervention and may have reduced the spread of some pathogens into latrine entrance soils.

Long-term cross-sectional follow-up study

We are revisiting MapSan trial compounds at least five years after the sanitation intervention to conduct a cross-sectional survey of children who were born after the intervention was implemented. Due to substantial population turnover [34], both intervention and control compounds are being identified using previously collected geolocation data and their locations confirmed by study staff during site identification visits. Any compounds identified in the study neighborhoods during the course of these mapping efforts that have received the sanitation intervention (built by WSUP to the same specifications) or match the original enrollment criteria for MapSan control compounds are also invited to participate, irrespective of previous participation in the MapSan trial. As an existing intervention, neither staff nor participants can be blinded to intervention status. We anticipate enrollment to continue for approximately one year and are again enrolling intervention and control compounds concurrently to limit any differential effects of seasonality and other secular trends across the enrollment period.

For peer review only - http://bmjopen.bmj.com/site/about/guidelines.xhtml

Patient and public involvement

Patients and/or the public were not involved in the design, conduct, reporting, or dissemination plans of this research.

Eligibility criteria

We attempt to enroll all eligible children in each compound that either previously participated in the MapSan trial, has previously received an intervention latrine, or meets the original enrollment criteria for MapSan control compounds [34]. Participant inclusion criteria include:

1. Child aged 29 days-60 months old
2. Born into and residing in a compound enrolled in the five-year follow-up study; in intervention compounds, the child must have been born following the delivery of the sanitation intervention
3. Has continuously resided in the study compound for the preceding 6 months, or since birth if under 6 months of age
4. Has a parent or guardian who is able to understand and complete the written informed consent process and allow their child to participate

Children are excluded if they have any caregiver-indicated medical condition or disability that precludes participation in the study.

Participant enrollment

Enrollment is conducted by trained study staff in either Portuguese or Changana, according to the respondent's preference, with written materials provided in Portuguese. We first obtain

For peer review only - http://bmjopen.bmj.com/site/about/guidelines.xhtml

verbal consent from the compound leader to approach households in the compound for enrollment. We then seek written, informed consent to participate from the parent or guardian of each eligible child. Participation is entirely voluntary; guardians may decline their child's participation for any reason and can withdraw their child at any point. We began enrolling participants on 28 March 2022.

Household visits and procedures

After locating study compounds, the household of a participating child is visited twice, typically on consecutive days. On the first day, trained study staff conduct written consent procedures; administer compound, household, and child questionnaires; record child anthropometry measures; collect environmental samples; and request the child's caregiver to retain a sample of the child's stool. The household is revisited the following day to collect a stool sample from the child and complete environmental sample collection. If the stool sample is unavailable, the study staff coordinate an additional visit to retrieve the stool. In the event that 7 days pass since the initial visit without collection of a stool sample, a qualified nurse visits the child to obtain a rectal swab.

After collection of the stool sample, deworming is offered to all household members >1 year old who have not been dewormed in the past year, unless pregnant or breastfeeding. Deworming consultation and medication provision is conducted by Ministry of Health staff following the national guidelines for deworming procedures. Deworming is offered in-kind to all household members and leverages the household interaction to provide an important public health service. Besides deworming, this study offers no direct benefit to children participating in this study. No

For peer review only - http://bmjopen.bmj.com/site/about/guidelines.xhtml

incentives are provided to study participants, but we provide 50 meticais (approximately US$1) of mobile phone credit on the caregiver's preferred network for each child to compensate for the costs incurred in communicating with the study team to arrange household visits. Households from which we collect food and/or large-volume water samples are reimbursed 50 additional meticais to offset these expenses.

Environmental sample collection

We are sampling environmental compartments at a randomly selected subset of 100 intervention and 100 control compounds to represent compound- and household-level exposures [38]. At the entrance to the compound latrine we collect soil, flies, and a large volume air sample, as well as fecal sludge from the latrine or septic tank and any animal feces observed in the shared outdoor space [50,51,57,58]. One household is randomly selected among those households with children enrolled in the child health study, from which we collect swabs of flooring at the household entrance, flies in cooking area, prepared child's food, stored drinking water, and water from the household's primary source [53,59,60].

We are also collecting environmental samples from the public domain, including wastewater, open surface water, soils in the vicinity of solid and fecal waste disposal locations, and wastewater effluent from hospitals treating COVID-19 patients. These samples are not linked to specific individuals in any way and are used to conduct environmental surveillance of pathogens circulating in the community [57,61].

For peer review only - http://bmjopen.bmj.com/site/about/guidelines.xhtml

Study outcomes

Enteric pathogen detection in stool

Stool-based molecular detection is performed for 27 enteric pathogens commonly implicated in both symptomatic and asymptomatic childhood infections globally, including those identified at the Global Enteric Multicenter Study (GEMS) study site in Manhiça, Mozambique [12,37,57,62]. Reverse-transcription quantitative polymerase chain reaction (RT-qPCR) is conducted by custom TaqMan Array Card (TAC; Thermo Fisher, Carlsbad, CA, USA) to simultaneously quantify genetic targets corresponding to 13 bacterial pathogens (*Aeromonas* spp.; *Campylobacter jejuni*/*coli*; *Clostridioides difficile*; *E. coli* O157; enteroaggregative *E. coli* (EAEC); enteropathogenic *E. coli* (EPEC); enterotoxigenic *E. coli* (ETEC); Shiga toxin-producing *E. coli* (STEC); enteroinvasive *E. coli* (EIEC)/*Shigella* spp.; *Helicobacter pylori*; *Plesiomonas shigelloides*; *Salmonella enterica*; *Vibrio cholerae*), 4 protozoan parasites (*Cryptosporidium* spp.; *Cyclospora cayetanensis*; *Entamoeba histolytica; Giardia* spp.), 5 soil transmitted helminths (*Ascaris lumbricoides*; *Ancylostoma duodenale*; *Necator americanus*; *Strongyloides stercolaris*; *Trichuris trichiura*), and 5 enteric viruses (adenovirus 40/41; astrovirus; norovirus GI/GII; rotavirus; sapovirus) [63]. We also include the respiratory virus SARS-CoV-2 on the custom TAC to support surveillance through fecal waste streams [64,65].

Our primary outcome is the prevalence of a pre-specified subset of 22 enteric pathogens; as in the original MapSan study, we exclude enteric viruses from the primary outcome due to the potential for direct contact transmission, which is unlikely to be impacted by the intervention [34,48,66]. Because we anticipate a combined prevalence (detection of at least one pathogen in a given stool sample) near 100%, and the intervention could plausibly increase the prevalence of

For peer review only - http://bmjopen.bmj.com/site/about/guidelines.xhtml

some pathogens while reducing others, we do not define a composite prevalence outcome [34,67]. Rather, we use mixed effects models to analyze all pathogens concurrently, thereby estimating the pooled effect of the intervention on enteric pathogen prevalence across the outcome set [68,69].

Secondary outcomes include the individual prevalence of all 27 pre-specified enteric pathogens, including *Shigella* spp. and *T. trichiura*, the two pathogens most impacted at 24 months among children born after the intervention was delivered [34]. A similar mixed effects model is used to analyze all pathogens concurrently to estimate separate treatment effects on the prevalence of each pathogen while accounting for correlations between outcomes, which alleviates concerns about *post-hoc* multiple comparisons adjustments [10,68]. Additional secondary outcomes include the continuous gene copy density in stool of the 22 primary outcome pathogens, for which a pooled treatment effect on the mean gene copy density is estimated, as well as the individual gene copy densities for all 27 enteric pathogens assessed on the TAC. Recognizing the potential challenges to interpretation due to pervasive exposure, persistent infection and rapid re-infection, and possible protective effects [70–73], we will also repeat the primary outcome analysis excluding *Giardia* from the outcome set.

Anthropometry

Child weight and recumbent length (child age < 24 months) or standing height (24-60 months) are assessed according to standard World Health Organization (WHO) protocols and transformed to age-adjusted z-scores using WHO reference populations to obtain height-for-age (HAZ), weight-for-age (WAZ), and weight-for-height (WHZ) z-scores [74,75]. Secondary outcomes

For peer review only - http://bmjopen.bmj.com/site/about/guidelines.xhtml

include continuous HAZ, WAZ, and WHZ, as well as prevalence of binary growth outcomes stunting (HAZ < -2), underweight (WAZ < -2), and wasting (WHZ < -2) [35].

Caregiver reported illness

Caregiver surveys are administered to ascertain child diarrheal disease, defined as the passage of three or more loose or watery stools in a 24-hour period, or any bloody stool, in the past 7 days [76]. We also assess two caregiver-reported negative control outcomes for each child: the 7-day period-prevalence of bruises, scrapes, or abrasions and the 7-day period-prevalence of toothache [77]. We do not expect the intervention to impact either child bruising or toothache prevalence, so significant differences in these outcomes by treatment arm would suggest possible bias in our caregiver-reported outcomes.

Pathogen detection in environmental matrices

Molecular detection of selected pathogens and fecal source tracking (FST) markers is performed for environmental samples from both the private (compound and household) and public domains using a second custom TAC [50,57,78,79]. A subset of the enteric pathogens assessed in stool is included on the environmental TAC (adenovirus 40/41; astrovirus; norovirus GI/GII; rotavirus; sapovirus; *Aeromonas* spp.; *C. jejuni*/*coli*; *C. difficile*; *E. coli* O157; EAEC; EPEC; ETEC; STEC; EIEC/*Shigella* spp.; *H. pylori*; *S. enterica*; *V. cholerae; Cryptosporidium* spp.; *E. histolytica; Giardia* spp.; *A. lumbricoides*; *A. duodenale*; *N. americanus*; *T. trichiura*), as well as select environmental and zoonotic pathogens (*Leptospira* spp; *Toxocara* spp.) [54,80,81], other human pathogens detectable in feces (SARS-CoV-2, Zika virus, HIV proviral DNA; *Plasmodium* spp.; *Mycobacterium tuberculosis*) [82–85], FST markers (human, poultry, and

For peer review only

For peer review only - http://bmjopen.bmj.com/site/about/guidelines.xhtml

canine mitochondrial DNA; avian 16S rRNA) [86,87], and general bacterial and anthropogenic pollution/antimicrobial resistance markers (bacterial 16S rRNA; class 1 integron-integrase gene *intI1*) [88,89]. We also culture fecal indicator bacteria (total coliforms and *E. coli*) using the IDEXX Colilert-18 and Quanti-Tray 2000 system [87,90]. We conduct ongoing genomic surveillance of SARS-CoV-2 viral lineages in wastewater samples using amplicon-based Illumina next generation sequencing [91].

Statistical analysis

Enteric pathogen outcomes

We will use mixed effects models with varying slopes and intercepts to estimate the weighted-average intervention effect across all pathogens included in the model, in essence treating each pathogen as a separate study of the intervention effect on enteric pathogen detection. Additional random effects will be included to account for clustering by compound and child. The prevalence odds ratio (POR) for children living in intervention compounds, relative to children in control compounds, will be estimated as the measure of effect for binary enteric pathogen detection outcomes, including the primary outcome and secondary individual pathogen detection outcomes. Prevalence differences (PD) between children in intervention and control compounds will also be estimated from the posterior predictive distribution at representative values of other model covariates [92]. Mean differences in standard deviation-scaled gene copy density will be estimated as the measure of effect for continuous enteric pathogen outcomes, with non-detects considered true zeros and censoring used to create a zero class (as in Tobit regression) [93]. Effect estimates will be summarized using the mean to represent the expected effect size and the central 95% probability interval to capture the range of effect sizes compatible with the data (the

For peer review only - http://bmjopen.bmj.com/site/about/guidelines.xhtml

95% CI). Parameters with 95% CIs that exclude the null will be considered significant, although the magnitude and uncertainty of parameter estimates will also be considered holistically in evaluating evidence for clinically meaningful effects [94].

Growth and caregiver reported outcomes

The effects of the intervention on mean HAZ, WAZ, and WHZ; the prevalence of stunting, underweight, and wasting; and the period-prevalence of caregiver-reported diarrhea and negative control outcomes (bruising, scrapes, and abrasions; toothache) will be analyzed separately as secondary outcomes using generalized estimating equations (GEE) and robust standard errors with exchangeable correlation structure and clustering by compound (the level at which the sanitation intervention was delivered) [34,35,95]. The estimated difference in age-adjusted z-scores by treatment assignment will be used as the measure of effect for continuous anthropometry outcomes. The prevalence ratio (PR) will be estimated by Poisson regression for binary growth status and caregiver-reported outcomes. We will not adjust for multiple comparisons [31,96].

Covariates and effect measure modification

As a cross-sectional study of an existing intervention, all analyses will be adjusted for a set of covariates selected *a priori* as potential confounders of the sanitation-enteric pathogen carriage relationship [97]. The adjustment set will include child age and sex, caregiver's education, and household wealth index [34,98]; records missing covariate data will be excluded from primary analyses. Analyses will be repeated with missing covariate data imputed by multivariate imputation using chained equations (MICE) as a sensitivity analysis [34,99]. Additional

For peer review only - http://bmjopen.bmj.com/site/about/guidelines.xhtml

covariates will be considered in exploratory adjusted analyses [10,11]. The specific enteric pathogens detected are expected to be strongly related to child age [8,34]. We will examine effect measure modification of the primary and secondary outcomes stratifying by age group (1-11 months, 12-23 months, and 24-60 months) [3,100].

Independently upgraded controls

We anticipate some of the control compounds may have independently upgraded their sanitation facilities to conditions comparable to the intervention. Control compounds with sanitation facilities observed to possess cleanable, intact hardscape slabs; pour-flush or water-sealed toilets; a functional ventilation pipe; and a fixed superstructure with sturdy walls and a secure door that ensure privacy during use are considered to have independently upgraded to conditions comparable to the intervention [56]. Children living in control compounds with independently upgraded latrines are enrolled but will be excluded from the main analyses of the intervention effects. Two sets of subgroup analyses will instead be conducted that include all participants: one in which children in independently upgraded controls are considered as part of the control (non-intervention) arm, and again considered as part of the intervention arm. We will compare parameter estimates from the three sets of analyses to investigate whether the sanitation improvements independently available in the study communities are comparable to the full sanitation intervention package assessed in the MapSan trial in terms of child health impacts.

Eligible children residing in any compound that has received a WSUP intervention latrine will be considered part of the intervention arm in primary analyses. We record the current conditions of intervention facilities but will not exclude or otherwise adjust for either upgraded or degraded

For peer review only - http://bmjopen.bmj.com/site/about/guidelines.xhtml

sanitation facilities in intervention compounds in order to evaluate the long-term impacts of the intervention following extended use.

Minimum detectable effect size

The number of participants will be constrained by the number of compounds in the study neighborhoods that have received the sanitation intervention or meet the eligibility requirements for MapSan control compounds, most of which were previously enrolled in the MapSan trial. At the 24 month follow-up, an average of 2.5 children per compound were enrolled from 408 compounds [34]. Compound-level intra-class correlation coefficients (ICCs) were generally less than 0.1 for individual pathogens, corresponding to cluster variances of ~0.05. We calculate the minimum detectable effect size (MDES) on individual pathogen prevalence with 80% power, 5% significance level, and 0.05 compound cluster variance for a conservative scenario with 200 compounds per treatment arm and 2 children enrolled per compound (for 800 children total, 400 per arm), a moderate scenario of 220 compounds per treatment arm and 2.5 children enrolled per compound (550 children per arm, 1100 total), and a maximal scenario of 300 compounds per arm, 2.5 children per compound (750 children per arm, 1500 total) [101]. Across all scenarios, a minimum baseline (untreated) prevalence of 6-8% is required to reach 80% power for even the largest theoretical effect (nearly 100% reduction). The minimum detectable relative reduction in prevalence decreases (that is, smaller effect sizes are more readily detected) as pathogen prevalence increases towards 100% (Figure 1). The difference between the scenarios on the multiplicative scale is relatively minor, with the relative reduction MDES largely driven by pathogen baseline prevalence. A pathogen with baseline prevalence below 15% must have its prevalence halved (PR < 0.5) in order to attain 80% power, while a 25% reduction is detectable

For peer review only - http://bmjopen.bmj.com/site/about/guidelines.xhtml

with 80% power for baseline prevalence of 34-46% under the maximal and conservative scenarios, respectively. We expect the simultaneous consideration of multiple pathogens to reduce the MDES for the primary outcome pooled intervention effect by effectively increasing the sample size. Because this pooled effect is dependent on the prevalence of each pathogen considered and the correlations between them, we will conduct simulation analyses to characterize plausible MDES ranges for the pooled primary outcome treatment effect [67,101].

For peer review only

For peer review only - http://bmjopen.bmj.com/site/about/guidelines.xhtml

LIMITATIONS

As an observational, cross-sectional evaluation of an existing intervention, this study faces a number of limitations that may impact the generalizability of our findings. By design, all of the study participants will have been born after the intervention was implemented in order to evaluate intervention impacts among individuals across a range of ages who have been exposed to intervention for their entire lives. However, there is potential for confounding bias in our estimates of the intervention impacts on child health, particularly concerning socioeconomic factors that may be associated with increased pathogen exposures. While non-randomized, the criteria for intervention and control compounds differed only by engineering considerations that we believe to be independent of the outcome. However, in the years since the intervention was implemented, the presence of the intervention itself may have influenced the desirability of the intervention sites and thus the socioeconomic status (SES) of their residents, particularly in light of the high population turnover previously observed after only 1-2 years. All analyses will be adjusted for a location-specific wealth index to account for potential differential SES between treatment arms [98], in addition to other pre-specified covariates associated with enteric pathogen exposure that may plausibly be related to treatment status, but the possibility of unmeasured and insufficiently controlled confounding remains.

Because childhood diarrhea is a leading cause of child mortality, selection bias arising from survivor effects may be present in our sample, particularly among older age groups [102]. However, loss to follow-up due to mortality was exceptionally rare in the previous assessment—far less common than emigration, which was not previously differential by treatment arm [34]. Our use of stool-based enteric pathogen detection as an objective primary outcome mitigates the

For peer review only - http://bmjopen.bmj.com/site/about/guidelines.xhtml

potential for measurement bias [37] and we are assessing multiple negative control outcomes to account for potential response bias in our secondary caregiver-reported outcomes [77]. As a long-term evaluation of an existing intervention, the potential for exposure measurement error is low—interventions that have degraded substantially may shift results towards the null, but in so doing would represent a valid assessment of intervention sustainability [103]. The potential for exposure misclassification among controls is higher, in that they may have independently upgraded to sanitation infrastructure comparable to the intervention. We are actively monitoring this possibility at all study sites; have pre-specified criteria for identifying sites achieving such conditions; and will conduct sensitivity analyses with these sites excluded, considered as controls, and considered as equivalent to the intervention to characterize the impact of any such potential exposure misclassification among controls.

A key challenge is identifying an appropriate set of outcomes against which to evaluate the intervention; in this regard, we preferred inclusivity given the pathogens likely to be observed in our study setting, at the risk of unduly shifting our eventual results towards the null. This challenge persists, regardless of study design, so long as the study aims include assessing the effect of some condition or intervention on exposure to, or infection by, multiple pathogens that share a common route of transmission [104].

CONTRIBUTION

Although observational, this study is unique in evaluating sanitation intervention effects at least five years after the intervention was implemented and up to five years after participants were borne into the intervention conditions. Previous studies have focused on WASH intervention

For peer review only - http://bmjopen.bmj.com/site/about/guidelines.xhtml

impacts up to 2-3 years after delivery [31–35], which may be insufficient time to realize impacts [43]. Furthermore, most previous studies have been conducted in rural settings, while we are investigating the long-term effects of an urban sanitation intervention that is broadly representative of the types of infrastructure improvements likely to be available in the rapidly growing urban informal settlements in coming years. Finally, we use stool-based detection of multiple enteric pathogens as an objective outcome and propose a novel pooled estimate of the treatment effect across a pre-specified outcome set to summarize the overall impact of the intervention on enteric pathogen exposure as the primary trial outcome.

For peer review only

For peer review only - http://bmjopen.bmj.com/site/about/guidelines.xhtml

ETHICS AND DISSEMINATION

This study was approved by Comité Nacional de Bioética para a Saúde, Ministério da Saúde de Moçambique (FWA#: 00003139, IRB00002657, 326/CNBS/21; approved: 15 June 2021) and University of North Carolina at Chapel Hill Ethics Committee (IRB#: 21-1119; approved 19 August 2021), and was prospectively registered with ISRCTN on 16 March 2022 (https://doi.org/10.1186/ISRCTN86084138). We pre-specified a statistical analysis plan that was deposited in a permanent online repository (https://osf.io/e7pvk/) prior to commencing enrollment. All protocol modification will be submitted to, and approved by, the respective ethics committees prior to implementation and promptly updated on the ISRCTN registry.

Written, informed consent is obtained from the parent(s)/guardian(s) of each participant. Identifiable information is maintained separately to protect confidentiality and linked to surveys, stool and environmental sample data by unique codes; the linking file to identifiable information will be destroyed upon completion of the study and the study data and remaining biospecimens retained for fully de-identified ancillary analyses. Results will be presented to key stakeholders in Mozambique, including local and national government officials, public utilities, and non-governmental organizations, and published in open access peer-reviewed journals. Upon publication of study results, the underlying individual participant data will be fully de-identified according to the Safe Harbor method and made freely available in the permanent online repository (https://osf.io/e7pvk/).

For peer review only

AUTHOR CONTRIBUTIONS

JB, EV, OC, RN, and JK designed the study, secured funding, and provided ongoing supervision. JB is also the corresponding author and guarantor. VM and DC drafted the initial study protocol and obtained ethical approvals. DH developed the statistical analysis plan, prepared the prospective trial registration and protocol manuscript, and is the first author. EK, GR, EM, JR, VA, AL, and YL developed and implemented the study procedures, which were overseen by VC, VM, MC, and NI. All authors contributed to and approved the final manuscript.

FUNDING

This work is funded by the Bill & Melinda Gates Foundation (OPP1137224) with additional support from a National Institute of Environmental Health Sciences training grant (T32ES007018). The funders had no role in the study design; data collection, analysis, and interpretation; or decision to publish.

COMPETING INTERESTS

The authors declare that they have no competing interests.

ACKNOWLEDGEMENTS

We thank all the participants, their families, and neighbors for graciously welcoming us into their communities, and our implementing partner, Water and Sanitation for the Urban Poor, for their continued support. We also gratefully acknowledge the hard work of the CISPOC survey team, including Jorge Binguane, Noémia Come, Anelsa Dunhe, Alice Fumo, Antônio Johane, Evelin

For peer review only - http://bmjopen.bmj.com/site/about/guidelines.xhtml

Matos, Eloisa Mula, Mariza Rachid, and Líria Sambo; and of Filipe Fazenda, Claúdia Machume, and Alfredo Muchanga in the CISPOC laboratory.

For peer review only

For peer review only - http://bmjopen.bmj.com/site/about/guidelines.xhtml

REFERENCES

1 Taniuchi M, Sobuz SU, Begum S, *et al.* Etiology of Diarrhea in Bangladeshi Infants in the First Year of Life Analyzed Using Molecular Methods. *Journal of Infectious Diseases* 2013;**208**:1794–802. doi:10.1093/infdis/jit507

2 Rogawski ET, Liu J, Platts-Mills JA, *et al.* Use of quantitative molecular diagnostic methods to investigate the effect of enteropathogen infections on linear growth in children in low-resource settings: longitudinal analysis of results from the MAL-ED cohort study. *The Lancet Global Health* 2018;**6**:e1319–28. doi:10.1016/S2214-109X(18)30351-6

3 Liu J, Platts-Mills JA, Juma J, *et al.* Use of quantitative molecular diagnostic methods to identify causes of diarrhoea in children: a reanalysis of the GEMS case-control study. *The Lancet* 2016;**388**:1291–301. doi:10.1016/S0140-6736(16)31529-X

4 Troeger CE, Khalil IA, Blacker BF, *et al.* Quantifying risks and interventions that have affected the burden of diarrhoea among children younger than 5 years: an analysis of the Global Burden of Disease Study 2017. *The Lancet Infectious Diseases* 2020;**20**:37–59. doi:10.1016/s1473-3099(19)30401-3

5 Black RE, Brown KH, Becker S. Effects of Diarrhea Associated with Specific Enteropathogens on the Growth of Children in Rural Bangladesh. *Pediatrics* 1984;**73**:799–805. doi:10.1542/peds.73.6.799

6 Benjamin-Chung J, Mertens A, Colford JM, *et al.* Early childhood linear growth faltering in low- and middle-income countries. medRxiv [preprint]. October 21, 2021 [cited 2022 Aug 30]. 2021. doi:10.1101/2020.06.09.20127001

7 Platts-Mills JA, Liu J, Rogawski ET, *et al.* Use of quantitative molecular diagnostic methods to assess the aetiology, burden, and clinical characteristics of diarrhoea in children in low-resource settings: a reanalysis of the MAL-ED cohort study. *The Lancet Global Health* 2018;**6**:e1309–18. doi:10.1016/S2214-109X(18)30349-8

8 Baker JM, Hasso-Agopsowicz M, Pitzer VE, *et al.* Association of enteropathogen detection with diarrhoea by age and high versus low child mortality settings: a systematic review and meta-analysis. *The Lancet Global Health* 2021;**9**:e1402–10. doi:10.1016/S2214-109X(21)00316-8

9 Schnee AE, Haque R, Taniuchi M, *et al.* Identification of Etiology-Specific Diarrhea Associated With Linear Growth Faltering in Bangladeshi Infants. *American Journal of Epidemiology* 2018;**187**:2210–8. doi:10.1093/aje/kwy106

10 Grembi JA, Lin A, Karim MA, *et al.* Effect of Water, Sanitation, Handwashing, and Nutrition Interventions on Enteropathogens in Children 14 Months Old: A Cluster-Randomized Controlled Trial in Rural Bangladesh. *The Journal of Infectious Diseases* Published Online First: August 2020. doi:10.1093/infdis/jiaa549

For peer review only - http://bmjopen.bmj.com/site/about/guidelines.xhtml

11 Rogawski McQuade ET, Platts-Mills JA, Gratz J, *et al.* Impact of Water Quality, Sanitation, Handwashing, and Nutritional Interventions on Enteric Infections in Rural Zimbabwe: The Sanitation Hygiene Infant Nutrition Efficacy (SHINE) Trial. *The Journal of Infectious Diseases* 2019;**221**:1379–86. doi:10.1093/infdis/jiz179

12 Knee J, Sumner T, Adriano Z, *et al.* Risk factors for childhood enteric infection in urban Maputo, Mozambique: A cross-sectional study. *PLOS Neglected Tropical Diseases* 2018;**12**:e0006956. doi:10.1371/journal.pntd.0006956

13 Lai AJ, Velez I, Ambikapathi R, *et al.* Independent and combined effects of nutrition and sanitation interventions on child growth and enteric pathogen infection in rural Cambodia: a factorial cluster-randomised controlled trial. medRxiv [preprint]. August 22, 2022 [cited August 30, 2022]. 2022. doi:10.1101/2021.05.21.21257546

14 Rego R, Watson S, Alam MAU, *et al.* A comparison of traditional diarrhoea measurement methods with microbiological and biochemical indicators: A cross-sectional observational study in the Cox's Bazar displaced persons camp. *eClinicalMedicine* 2021;**42**:101205. doi:10.1016/j.eclinm.2021.101205

15 Berendes D, Capone D, Knee J, *et al.* Associations between enteric pathogen carriage and height-for-age, weight-for-age and weight-for-height in children under 5 years old in urban Dhaka, Bangladesh. *Epidemiology and Infection* 2020;**148**:e39. doi:10.1017/s0950268820000369

16 Collard J-M, Andrianonimiadana L, Habib A, *et al.* High prevalence of small intestine bacteria overgrowth and asymptomatic carriage of enteric pathogens in stunted children in Antananarivo, Madagascar. *PLOS Neglected Tropical Diseases* 2022;**16**:1–22. doi:10.1371/journal.pntd.0009849

17 Garzón M, Pereira-da-Silva L, Seixas J, *et al.* Subclinical Enteric Parasitic Infections and Growth Faltering in Infants in São Tomé, Africa: A Birth Cohort Study. *International Journal of Environmental Research and Public Health* 2018;**15**. doi:10.3390/ijerph15040688

18 Prendergast AJ, Kelly P. Interactions between intestinal pathogens, enteropathy and malnutrition in developing countries. *Current opinion in infectious diseases* 2016;:1. doi:10.1097/QCO.0000000000000261

19 Prendergast AJ, Rukobo S, Chasekwa B, *et al.* Stunting Is Characterized by Chronic Inflammation in Zimbabwean Infants. *Plos One* 2014;**9**:11. doi:10.1371/journal.pone.0086928

20 George CM, Burrowes V, Perin J, *et al.* Enteric Infections in Young Children are Associated with Environmental Enteropathy and Impaired Growth. *Tropical Medicine & International Health* 2017;**12**:3218–21. doi:10.1111/tmi.13002

For peer review only - http://bmjopen.bmj.com/site/about/guidelines.xhtml

21 Kosek MN, Ahmed T, Bhutta Z, *et al.* Causal Pathways from Enteropathogens to Environmental Enteropathy: Findings from the MAL-ED Birth Cohort Study. *EBioMedicine* 2017;**18**:109–17. doi:10.1016/j.ebiom.2017.02.024

22 Hoddinott J, Behrman JR, Maluccio JA, *et al.* Adult consequences of growth failure in early childhood. *American Journal of Clinical Nutrition* 2013;**98**:1170–8. doi:10.3945/ajcn.113.064584

23 MAL-ED Network Investigators. Early childhood cognitive development is affected by interactions among illness, diet, enteropathogens and the home environment: findings from the MAL-ED birth cohort study. *BMJ Global Health* 2018;**3**:e000752. doi:10.1136/bmjgh-2018-000752

24 Oriá RB, Murray-Kolb LE, Scharf RJ, *et al.* Early-life enteric infections: relation between chronic systemic inflammation and poor cognition in children. *Nutrition Reviews* 2016;**74**:374–86. doi:10.1093/nutrit/nuw008

25 Donowitz JR, Drew J, Taniuchi M, *et al.* Diarrheal Pathogens Associated With Growth and Neurodevelopment. *Clinical Infectious Diseases* 2021;**73**:e683–91. doi:10.1093/cid/ciaa1938

26 Parker EP, Ramani S, Lopman BA, *et al.* Causes of impaired oral vaccine efficacy in developing countries. *Future Microbiology* 2018;**13**:97–118. doi:10.2217/fmb-2017-0128

27 Taniuchi M, Platts-Mills JA, Begum S, *et al.* Impact of enterovirus and other enteric pathogens on oral polio and rotavirus vaccine performance in Bangladeshi infants. *Vaccine* 2016;**34**:3068–75. doi:10.1016/j.vaccine.2016.04.080

28 Cumming O, Cairncross S. Can water, sanitation and hygiene help eliminate stunting? Current evidence and policy implications. *Maternal & Child Nutrition* 2016;**12**:91–105. doi:10.1111/mcn.12258

29 Wolf J, Hubbard S, Brauer M, *et al.* Effectiveness of interventions to improve drinking water, sanitation, and handwashing with soap on risk of diarrhoeal disease in children in low-income and middle-income settings: a systematic review and meta-analysis. *The Lancet* 2022;**400**:48–59. doi:10.1016/s0140-6736(22)00937-0

30 Contreras JD, Eisenberg JNS. Does Basic Sanitation Prevent Diarrhea? Contextualizing Recent Intervention Trials through a Historical Lens. *International Journal of Environmental Research and Public Health* 2020;**17**. doi:10.3390/ijerph17010230

31 Luby SP, Rahman M, Arnold BF, *et al.* Effects of water quality, sanitation, handwashing, and nutritional interventions on diarrhoea and child growth in rural Bangladesh: a cluster randomised controlled trial. *The Lancet Global Health* 2018;**6**:e302–15. doi:10.1016/s2214-109x(17)30490-4

32 Null C, Stewart CP, Pickering AJ, *et al.* Effects of water quality, sanitation, handwashing, and nutritional interventions on diarrhoea and child growth in rural Kenya: a cluster-

For peer review only

For peer review only - http://bmjopen.bmj.com/site/about/guidelines.xhtml

randomised controlled trial. *The Lancet Global Health* 2018;**6**:e316–29. doi:10.1016/s2214-109x(18)30005-6

33 Humphrey JH, Mbuya MNN, Ntozini R, *et al.* Independent and combined effects of improved water, sanitation, and hygiene, and improved complementary feeding, on child stunting and anaemia in rural Zimbabwe: a cluster-randomised trial. *The Lancet Global Health* 2019;**7**:e132–47. doi:10.1016/s2214-109x(18)30374-7

34 Knee J, Sumner T, Adriano Z, *et al.* Effects of an urban sanitation intervention on childhood enteric infection and diarrhea in Maputo, Mozambique: a controlled before-and-after trial. *eLife* 2021;**10**:e62278. doi:10.7554/elife.62278

35 Lai A, Velez I, Ambikapathi R, *et al.* Independent and combined effects of nutrition and sanitation interventions on child growth and enteric pathogen infection in rural Cambodia: a factorial cluster-randomised controlled trial. *In preparation* 2022.

36 Watson SI, Rego RTT, Hofer T, *et al.* Evaluations of water, sanitation and hygiene interventions should not use diarrhoea as (primary) outcome. *BMJ Global Health* 2022;**7**. doi:10.1136/bmjgh-2022-008521

37 Brown J, Cumming O. Stool-Based Pathogen Detection Offers Advantages as an Outcome Measure for Water, Sanitation, and Hygiene Trials. *The American Journal of Tropical Medicine and Hygiene* 2020;**102**:260–1. doi:10.4269/ajtmh.19-0639

38 Goddard F, Ban R, Barr DB, *et al.* Measuring environmental exposure to enteric pathogens in low-income settings: review and recommendations of an interdisciplinary working group. *Environ Sci Technol* 2020;:acs.est.0c02421. doi:10.1021/acs.est.0c02421

39 Ercumen A, Benjamin-Chung J, Arnold BF, *et al.* Effects of water, sanitation, handwashing and nutritional interventions on soil-transmitted helminth infections in young children: A cluster-randomized controlled trial in rural Bangladesh. *PLoS Neglected Tropical Diseases* 2019;**13**:1–24. doi:10.1371/journal.pntd.0007323

40 Lin A, Ercumen A, Benjamin-Chung J, *et al.* Effects of water, sanitation, handwashing, and nutritional interventions on child enteric protozoan infections in rural Bangladesh: A cluster-randomized controlled trial. *Clinical Infectious Diseases* 2018;**67**:1515–22. doi:10.1093/cid/ciy320

41 Pickering AJ, Njenga SM, Steinbaum L, *et al.* Effects of single and integrated water, sanitation, handwashing, and nutrition interventions on child soil-transmitted helminth and Giardia infections: A cluster-randomized controlled trial in rural Kenya. *PLOS Medicine* 2019;**16**:e1002841. doi:10.1371/journal.pmed.1002841

42 Fuhrmeister ER, Ercumen A, Pickering AJ, *et al.* Effect of sanitation improvements on pathogens and microbial source tracking markers in the rural Bangladeshi household environment. *Environ Sci Technol* 2020;:acs.est.9b04835. doi:10.1021/acs.est.9b04835

For peer review only - http://bmjopen.bmj.com/site/about/guidelines.xhtml

43 Pickering AJ, Null C, Winch PJ, *et al.* The WASH Benefits and SHINE trials: interpretation of WASH intervention effects on linear growth and diarrhoea. *The Lancet Global Health* 2019;**7**:e1139–46. doi:10.1016/s2214-109x(19)30268-2

44 Amebelu A, Ban R, Bhagwan J, *et al.* The Lancet Commission on water, sanitation and hygiene, and health. *The Lancet* 2021;**398**:1469–70. doi:10.1016/S0140-6736(21)02005-5

45 Budge S, Ambelu A, Bartram J, *et al.* Environmental sanitation and the evolution of water, sanitation and hygiene. *Bull World Health Organ* 2022;**100**:286–8. doi:10.2471/BLT.21.287137

46 Ezeh A, Oyebode O, Satterthwaite D, *et al.* The history, geography, and sociology of slums and the health problems of people who live in slums. *The Lancet* 2017;**389**:547–58. doi:10.1016/S0140-6736(16)31650-6

47 Sinharoy SS, Pittluck R, Clasen T. Review of drivers and barriers of water and sanitation policies for urban informal settlements in low-income and middle-income countries. *Utilities Policy* 2019;**60**:100957. doi:10.1016/j.jup.2019.100957

48 Brown J, Cumming O, Bartram J, *et al.* A controlled, before-and-after trial of an urban sanitation intervention to reduce enteric infections in children: research protocol for the Maputo Sanitation (MapSan) study, Mozambique. *BMJ Open* 2015;**5**:e008215–e008215. doi:10.1136/bmjopen-2015-008215

49 Bick S, Buxton H, Chase RP, *et al.* Using path analysis to test theory of change: a quantitative process evaluation of the MapSan trial. *BMC Public Health* 2021;**21**:1411. doi:10.1186/s12889-021-11364-w

50 Capone D, Berendes D, Cumming O, *et al.* Impact of an Urban Sanitation Intervention on Enteric Pathogen Detection in Soils. *Environ Sci Technol* 2021;**55**:9989–10000. doi:10.1021/acs.est.1c02168

51 Holcomb DA, Knee J, Capone D, *et al.* Impacts of an Urban Sanitation Intervention on Fecal Indicators and the Prevalence of Human Fecal Contamination in Mozambique. *Environ Sci Technol* 2021;**55**:11667–79. doi:10.1021/acs.est.1c01538

52 Fuhrmeister ER, Ercumen A, Pickering AJ, *et al.* Predictors of Enteric Pathogens in the Domestic Environment from Human and Animal Sources in Rural Bangladesh. *Environmental Science & Technology* 2019;**53**:10023–33. doi:10.1021/acs.est.8b07192

53 Holcomb DA, Knee J, Sumner T, *et al.* Human fecal contamination of water, soil, and surfaces in households sharing poor-quality sanitation facilities in Maputo, Mozambique. *International Journal of Hygiene and Environmental Health* 2020;**226**:113496. doi:10.1016/j.ijheh.2020.113496

54 Delahoy MJ, Wodnik B, McAliley L, *et al.* Pathogens Transmitted in Animal Feces in Low- and Middle-Income Countries. *International Journal of Hygiene and Environmental Health* 2018. doi:10.1016/j.ijheh.2018.03.005

For peer review only - http://bmjopen.bmj.com/site/about/guidelines.xhtml

55 Capone D, Adriano Z, Berendes D, *et al.* A localized sanitation status index as a proxy for fecal contamination in urban Maputo, Mozambique. *PLOS ONE* 2019;**14**:e0224333. doi:10.1371/journal.pone.0224333

56 Shiras T, Cumming O, Brown J, *et al.* Shared latrines in Maputo, Mozambique: exploring emotional well-being and psychosocial stress. *BMC International Health and Human Rights* 2018;**18**:30. doi:10.1186/s12914-018-0169-z

57 Capone D, Berendes D, Cumming O, *et al.* Analysis of Fecal Sludges Reveals Common Enteric Pathogens in Urban Maputo, Mozambique. *Environ Sci Technol Lett* 2020;:acs.estlett.0c00610. doi:10.1021/acs.estlett.0c00610

58 Ginn O, Rocha-Melogno L, Bivins A, *et al.* Detection and Quantification of Enteric Pathogens in Aerosols Near Open Wastewater Canals in Cities with Poor Sanitation. *Environ Sci Technol* 2021;**55**:14758–71. doi:10.1021/acs.est.1c05060

59 Bick S, Perieres L, D'Mello-Guyett L, *et al.* Risk factors for child food contamination in low-income neighbourhoods of Maputo, Mozambique: An exploratory, cross-sectional study. *Maternal & Child Nutrition* 2020;:e12991. doi:10.1111/mcn.12991

60 Exum NG, Olórtegui MP, Yori PP, *et al.* Floors and Toilets: Association of Floors and Sanitation Practices with Fecal Contamination in Peruvian Amazon Peri-Urban Households. *Environmental Science and Technology* 2016;**50**:7373–81. doi:10.1021/acs.est.6b01283

61 Schang C, Crosbie ND, Nolan M, *et al.* Passive Sampling of SARS-CoV-2 for Wastewater Surveillance. *Environ Sci Technol* 2021;**55**:10432–41. doi:10.1021/acs.est.1c01530

62 Kotloff KL, Nataro JP, Blackwelder WC, *et al.* Burden and aetiology of diarrhoeal disease in infants and young children in developing countries (the Global Enteric Multicenter Study, GEMS): A prospective, case-control study. *The Lancet* 2013;**382**:209–22. doi:10.1016/s0140-6736(13)60844-2

63 Liu J, Gratz J, Amour C, *et al.* Optimization of Quantitative PCR Methods for Enteropathogen Detection. *PLOS ONE* 2016;**11**:e0158199. doi:10.1371/journal.pone.0158199

64 Natarajan A, Zlitni S, Brooks EF, *et al.* Gastrointestinal symptoms and fecal shedding of SARS-CoV-2 RNA suggest prolonged gastrointestinal infection. *Med* 2022;**3**:371-387.e9. doi:10.1016/j.medj.2022.04.001

65 Bivins A, North D, Ahmad A, *et al.* Wastewater-Based Epidemiology: Global Collaborative to Maximize Contributions in the Fight Against COVID-19. *Environ Sci Technol* 2020;:acs.est.0c02388. doi:10.1021/acs.est.0c02388

66 Wikswo ME, Kambhampati A, Shioda K, *et al.* Outbreaks of Acute Gastroenteritis Transmitted by Person-to-Person Contact, Environmental Contamination, and Unknown

For peer review only - http://bmjopen.bmj.com/site/about/guidelines.xhtml

Modes of Transmission — United States, 2009–2013. *MMWR Surveill Summ* 2015;**64**:1–16. doi:10.15585/mmwr.ss6412a1

67 Vickerstaff V, Ambler G, Omar RZ. A comparison of methods for analysing multiple outcome measures in randomised controlled trials using a simulation study. *Biometrical Journal* 2021;**63**:599–615. doi:10.1002/bimj.201900040

68 Gelman A, Hill J, Yajima M. Why We (Usually) Don't Have to Worry About Multiple Comparisons. *Journal of Research on Educational Effectiveness* 2012;**5**:189–211. doi:10.1080/19345747.2011.618213

69 Mitchell S, Gelman A, Ross R, *et al.* The Millennium Villages Project: a retrospective, observational, endline evaluation. *The Lancet Global Health* 2018;**6**:e500–13. doi:10.1016/s2214-109x(18)30065-2

70 Donowitz JR, Alam M, Kabir M, *et al.* A Prospective Longitudinal Cohort to Investigate the Effects of Early Life Giardiasis on Growth and All Cause Diarrhea. *Clinical Infectious Diseases* 2016;**63**:792–7. doi:10.1093/cid/ciw391

71 Bartelt LA, Platts-Mills JA. Giardia: a pathogen or commensal for children in high-prevalence settings? *Current Opinion in Infectious Diseases* 2016;**29**:502–7. doi:10.1097/qco.0000000000000293

72 Rogawski ET, Bartelt LA, Platts-Mills JA, *et al.* Determinants and Impact of Giardia Infection in the First 2 Years of Life in the MAL-ED Birth Cohort. *Journal of the Pediatric Infectious Diseases Society* 2017;**6**:153–60. doi:10.1093/jpids/piw082

73 Messa A, Köster PC, Garrine M, *et al.* Molecular diversity of Giardia duodenalis in children under 5 years from the Manhiça district, Southern Mozambique enrolled in a matched case-control study on the aetiology of diarrhoea. *PLoS Negl Trop Dis* 2021;**15**:e0008987. doi:10.1371/journal.pntd.0008987

74 de Onis M, Garza C, Victora CG, *et al.* The WHO Multicentre Growth Reference Study: Planning, Study Design, and Methodology. *Food Nutr Bull* 2004;**25**:S15–26. doi:10.1177/15648265040251S104

75 WHO. *Length/height-for-age, weight-for-age, weight-for-length, weight-for-height and body mass index-for-age: Methods and development*. Geneva: : World Health Organization 2006.

76 Arnold BF, Galiani S, Ram PK, *et al.* Optimal Recall Period for Caregiver-reported Illness in Risk Factor and Intervention Studies: A Multicountry Study. *American Journal of Epidemiology* 2013;**177**:361–70. doi:10.1093/aje/kws281

77 Arnold BF, Ercumen A, Benjamin-Chung J, *et al.* Brief Report: Negative Controls to Detect Selection Bias and Measurement Bias in Epidemiologic Studies. *Epidemiology* 2016;**27**:637–41. doi:10.1097/EDE.0000000000000504

For peer review only - http://bmjopen.bmj.com/site/about/guidelines.xhtml

78 Lappan R, Henry R, Chown SL, *et al.* Monitoring of diverse enteric pathogens across environmental and host reservoirs with TaqMan array cards and standard qPCR: a methodological comparison study. *The Lancet Planetary Health* 2021;**5**:e297–308. doi:10.1016/S2542-5196(21)00051-6

79 Baker KK, Senesac R, Sewell D, *et al.* Fecal Fingerprints of Enteric Pathogen Contamination in Public Environments of Kisumu, Kenya, Associated with Human Sanitation Conditions and Domestic Animals. *Environmental Science & Technology* 2018;**52**:10263–74. doi:10.1021/acs.est.8b01528

80 Rostami A, Ma G, Wang T, *et al.* Human toxocariasis – A look at a neglected disease through an epidemiological 'prism.' *Infection, Genetics and Evolution* 2019;**74**:104002. doi:10.1016/j.meegid.2019.104002

81 Schneider AG, Casanovas-Massana A, Hacker KP, *et al.* Quantification of pathogenic Leptospira in the soils of a Brazilian urban slum. *PLOS Neglected Tropical Diseases* 2018;**12**:1–15. doi:10.1371/journal.pntd.0006415

82 Muirhead A, Zhu K, Brown J, *et al.* Zika Virus RNA Persistence in Sewage. *Environ Sci Technol Lett* 2020;**7**:659–64. doi:10.1021/acs.estlett.0c00535

83 Chakrabarti AK, Caruso L, Ding M, *et al.* Detection of HIV-1 RNA/DNA and CD4 mRNA in feces and urine from chronic HIV-1 infected subjects with and without anti-retroviral therapy. *AIDS Res Ther* 2009;**6**:20. doi:10.1186/1742-6405-6-20

84 Jirků M, Pomajbíková K, Petrželková KJ, *et al.* Detection of *Plasmodium* spp. in Human Feces. *Emerg Infect Dis* 2012;**18**. doi:10.3201/eid1804.110984

85 Sultana S, Ansar A, Saif-Ur-Rahman KM. Stool specimen for diagnosis of pulmonary tuberculosis in adults: protocol for a systematic review and meta-analysis. *BMJ Open* 2021;**11**:e052212. doi:10.1136/bmjopen-2021-052212

86 Zhu K, Suttner B, Pickering A, *et al.* A novel droplet digital PCR human mtDNA assay for fecal source tracking. *Water Research* 2020;:116085. doi:10.1016/j.watres.2020.116085

87 Holcomb DA, Stewart JR. Microbial Indicators of Fecal Pollution: Recent Progress and Challenges in Assessing Water Quality. *Curr Envir Health Rpt* 2020;**7**:311–24. doi:10.1007/s40572-020-00278-1

88 Gillings MR, Gaze WH, Pruden A, *et al.* Using the class 1 integron-integrase gene as a proxy for anthropogenic pollution. *The ISME Journal* 2015;**9**:1269–79. doi:10.1038/ismej.2014.226

89 Keenum I, Liguori K, Calarco J, *et al.* A framework for standardized qPCR-targets and protocols for quantifying antibiotic resistance in surface water, recycled water and wastewater. *null* 2022;:1–25. doi:10.1080/10643389.2021.2024739

For peer review only - http://bmjopen.bmj.com/site/about/guidelines.xhtml

90 Julian TR, Islam MA, Pickering AJ, *et al.* Genotypic and Phenotypic Characterization of Escherichia coli Isolates from Feces, Hands, and Soils in Rural Bangladesh via the Colilert Quanti-Tray System. *Appl Environ Microbiol* 2015;**81**:1735–43. doi:10.1128/AEM.03214-14

91 Karthikeyan S, Levy JI, De Hoff P, *et al.* Wastewater sequencing reveals early cryptic SARS-CoV-2 variant transmission. *Nature* Published Online First: 7 July 2022. doi:10.1038/s41586-022-05049-6

92 Gelman A, Pardoe I. 2. Average Predictive Comparisons for Models with Nonlinearity, Interactions, and Variance Components. *Sociological Methodology* 2007;**37**:23–51. doi:10.1111/j.1467-9531.2007.00181.x

93 Clark JS, Nemergut D, Seyednasrollah B, *et al.* Generalized joint attribute modeling for biodiversity analysis: median-zero, multivariate, multifarious data. *Ecological Monographs* 2017;**87**:34–56. doi:10.1002/ecm.1241

94 Rafi Z, Greenland S. Semantic and cognitive tools to aid statistical science: replace confidence and significance by compatibility and surprise. *BMC Medical Research Methodology* 2020;**20**:244. doi:10.1186/s12874-020-01105-9

95 Bottomley C, Kirby MJ, Lindsay SW, *et al.* Can the buck always be passed to the highest level of clustering? *BMC Medical Research Methodology* 2016;**16**:29. doi:10.1186/s12874-016-0127-1

96 Rothman KJ. No Adjustments Are Needed for Multiple Comparisons. *Epidemiology* 1990;**1**. doi:10.1097/00001648-199001000-00010

97 VanderWeele TJ. Principles of confounder selection. *Eur J Epidemiol* 2019;**34**:211–9. doi:10.1007/s10654-019-00494-6

98 Schreiner M. Simple Poverty Scorecard Poverty-Assessment Tool Mozambique. Microfinance Risk Management, LLC 2017. https://www.simplepovertyscorecard.com/MOZ_2014_ENG.pdf

99 Buuren S van, Groothuis-Oudshoorn K. mice: Multivariate Imputation by Chained Equations in R. *Journal of Statistical Software* 2011;**45**. doi:10.18637/jss.v045.i03

100 VanderWeele TJ, Knol MJ. A Tutorial on Interaction. *Epidemiologic Methods* 2014;**3**:33–72. doi:10.1515/em-2013-0005

101 Arnold BF, Hogan DR, Colford JMJ, *et al.* Simulation methods to estimate design power: an overview for applied research. *BMC Medical Research Methodology* 2011;**11**:94. doi:10.1186/1471-2288-11-94

102 Hernán MA, Hernández-Díaz S, Robins JM. A Structural Approach to Selection Bias. *Epidemiology* 2004;**15**:615–25. doi:10.1097/01.ede.0000135174.63482.43

For peer review only - http://bmjopen.bmj.com/site/about/guidelines.xhtml

103 Goddard FGB, Chang HH, Clasen TF, *et al.* Exposure measurement error and the characterization of child exposure to fecal contamination in drinking water. *npj Clean Water* 2020;**3**:19. doi:10.1038/s41545-020-0063-9

104 Lash TL, Fox MP, Maclehose RF, *et al.* Good practices for quantitative bias analysis. *International Journal of Epidemiology* 2014;**43**:1969–85. doi:10.1093/ije/dyu149

For peer review only

For peer review only - http://bmjopen.bmj.com/site/about/guidelines.xhtml

FIGURE CAPTION

Figure 1. Range of minimum detectable effect sizes (shaded region) for the percent reduction in pathogen prevalence with 80% power, 5% significance level, and 0.05 cluster variance across three sample size scenarios. The upper edge of the shaded area represents a conservative scenario with 800 total participants (2 per compound, 200 compounds per arm) while the lower edge corresponds to a maximal scenario with 1500 total participants (2.5 per compound, 300 compounds per arm). A moderate scenario with 1100 total participants (2.5 per compound, 220 compounds per arm) is represented by the black curve within the shaded area. The vertical lines show the prevalence of a subset of pathogens assessed in control compound children during the 24-month follow-up in the original MapSan trial. Line color indicates the specific pathogen and line pattern reflects the pathogen class.

For peer review only

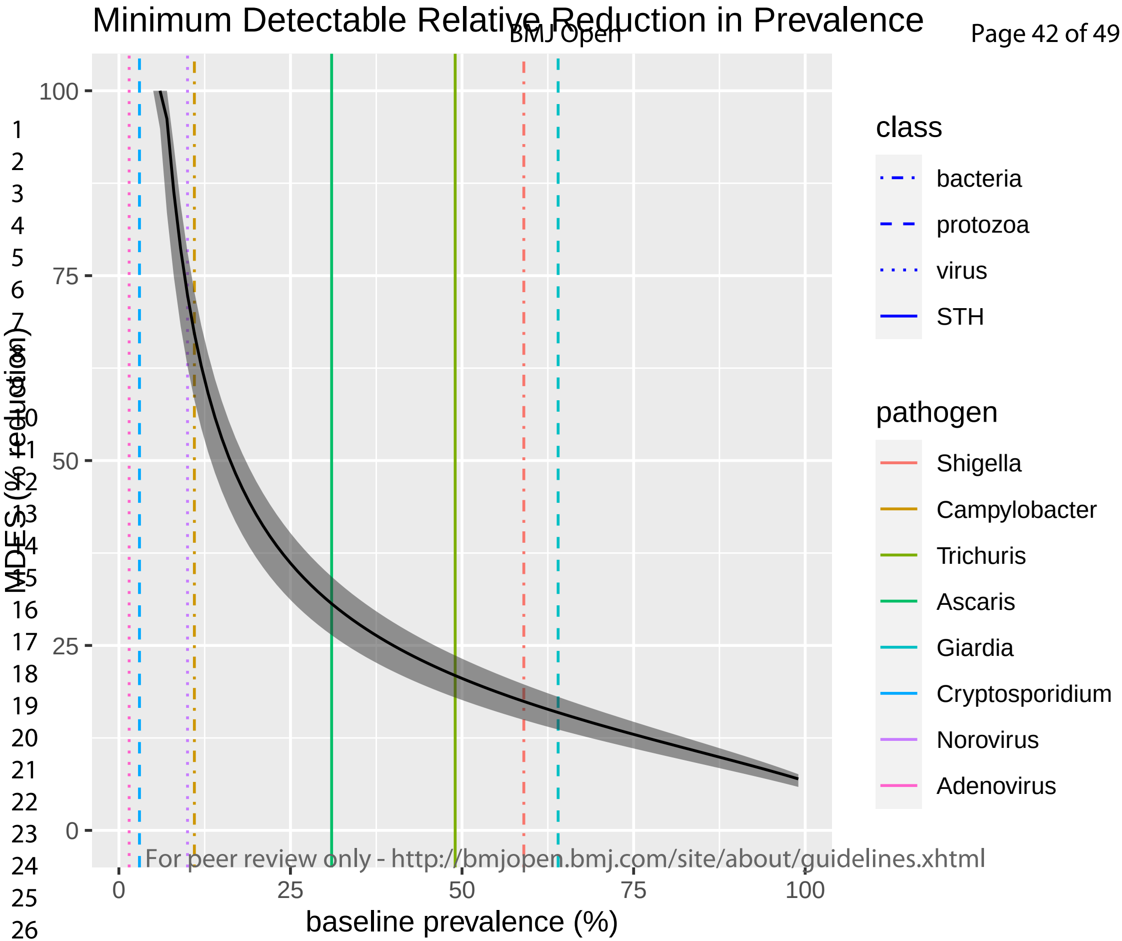

For peer review only - http://bmjopen.bmj.com/site/about/guidelines.xhtml

Table S1. SPIRIT Checklist

		Reporting Item	Page Number
**Administrative information**			
Title	#1	Descriptive title identifying the study design, population, interventions, and, if applicable, trial acronym	1
Trial registration	#2a	Trial identifier and registry name. If not yet registered, name of intended registry	27
Trial registration: data set	#2b	All items from the World Health Organization Trial Registration Data Set	Table S2
Protocol version	#3	Date and version identifier	NA
Funding	#4	Sources and types of financial, material, and other support	28
Roles and responsibilities: contributorship	#5a	Names, affiliations, and roles of protocol contributors	1, 28
Roles and responsibilities: sponsor contact information	#5b	Name and contact information for the trial sponsor	n/a
Roles and responsibilities: sponsor and funder	#5c	Role of study sponsor and funders, if any, in study design; collection, management, analysis, and interpretation of data; writing of the report; and the decision to submit the report for publication, including whether they will have ultimate authority over any of these activities	28
Roles and responsibilities: committees	#5d	Composition, roles, and responsibilities of the coordinating centre, steering committee, endpoint adjudication committee, data management team, and other individuals or groups overseeing the trial, if applicable (see Item 21a for data monitoring committee)	n/a

For peer review only - http://bmjopen.bmj.com/site/about/guidelines.xhtml

		Reporting Item	Page Number
**Introduction**			
Background and rationale	#6a	Description of research question and justification for undertaking the trial, including summary of relevant studies (published and unpublished) examining benefits and harms for each intervention	6-8
Background and rationale: choice of comparators	#6b	Explanation for choice of comparators	11
Objectives	#7	Specific objectives or hypotheses	9
Trial design	#8	Description of trial design including type of trial (eg, parallel group, crossover, factorial, single group), allocation ratio, and framework (eg, superiority, equivalence, non-inferiority, exploratory)	12
**Methods: Participants, interventions, and outcomes**			
Study setting	#9	Description of study settings (eg, community clinic, academic hospital) and list of countries where data will be collected. Reference to where list of study sites can be obtained	10
Eligibility criteria	#10	Inclusion and exclusion criteria for participants. If applicable, eligibility criteria for study centres and individuals who will perform the interventions (eg, surgeons, psychotherapists)	13
Interventions: description	#11a	Interventions for each group with sufficient detail to allow replication, including how and when they will be administered	10-11
Interventions: modifications	#11b	Criteria for discontinuing or modifying allocated interventions for a given trial participant (eg, drug dose change in response to harms, participant request, or improving / worsening disease)	n/a
Interventions: adherance	#11c	Strategies to improve adherence to intervention protocols, and any procedures for monitoring adherence (eg, drug tablet return; laboratory tests)	n/a

For peer review only - http://bmjopen.bmj.com/site/about/guidelines.xhtml

		Reporting Item	Page Number
Interventions: concomitant care	#11d	Relevant concomitant care and interventions that are permitted or prohibited during the trial	21-22
Outcomes	#12	Primary, secondary, and other outcomes, including the specific measurement variable (eg, systolic blood pressure), analysis metric (eg, change from baseline, final value, time to event), method of aggregation (eg, median, proportion), and time point for each outcome. Explanation of the clinical relevance of chosen efficacy and harm outcomes is strongly recommended	16-18
Participant timeline	#13	Time schedule of enrolment, interventions (including any run-ins and washouts), assessments, and visits for participants.	13-14
Sample size	#14	Estimated number of participants needed to achieve study objectives and how it was determined, including clinical and statistical assumptions supporting any sample size calculations	22-23
Recruitment	#15	Strategies for achieving adequate participant enrolment to reach target sample size	12-13
**Methods: Assignment of interventions (for controlled trials)**			
Allocation: sequence generation	#16a	Method of generating the allocation sequence (eg, computer-generated random numbers), and list of any factors for stratification. To reduce predictability of a random sequence, details of any planned restriction (eg, blocking) should be provided in a separate document that is unavailable to those who enrol participants or assign interventions	n/a
Allocation concealment mechanism	#16b	Mechanism of implementing the allocation sequence (eg, central telephone; sequentially numbered, opaque, sealed envelopes), describing any steps to conceal the sequence until interventions are assigned	n/a
Allocation: implementation	#16c	Who will generate the allocation sequence, who will enrol participants, and who will assign participants to interventions	n/a

For peer review only

		Reporting Item	Page Number
Blinding (masking)	#17a	Who will be blinded after assignment to interventions (eg, trial participants, care providers, outcome assessors, data analysts), and how	n/a
Blinding (masking): emergency unblinding	#17b	If blinded, circumstances under which unblinding is permissible, and procedure for revealing a participant's allocated intervention during the trial	n/a
**Methods: Data collection, management, and analysis**			
Data collection plan	#18a	Plans for assessment and collection of outcome, baseline, and other trial data, including any related processes to promote data quality (eg, duplicate measurements, training of assessors) and a description of study instruments (eg, questionnaires, laboratory tests) along with their reliability and validity, if known. Reference to where data collection forms can be found, if not in the protocol	14
Data collection plan: retention	#18b	Plans to promote participant retention and complete follow-up, including list of any outcome data to be collected for participants who discontinue or deviate from intervention protocols	n/a
Data management	#19	Plans for data entry, coding, security, and storage, including any related processes to promote data quality (eg, double data entry; range checks for data values). Reference to where details of data management procedures can be found, if not in the protocol	27
Statistics: outcomes	#20a	Statistical methods for analysing primary and secondary outcomes. Reference to where other details of the statistical analysis plan can be found, if not in the protocol	19-20
Statistics: additional analyses	#20b	Methods for any additional analyses (eg, subgroup and adjusted analyses)	21-22

For peer review only

		Reporting Item	Page Number
Statistics: analysis population and missing data	#20c	Definition of analysis population relating to protocol non-adherence (eg, as randomised analysis), and any statistical methods to handle missing data (eg, multiple imputation)	21
**Methods: Monitoring**			
Data monitoring: formal committee	#21a	Composition of data monitoring committee (DMC); summary of its role and reporting structure; statement of whether it is independent from the sponsor and competing interests; and reference to where further details about its charter can be found, if not in the protocol. Alternatively, an explanation of why a DMC is not needed	n/a
Data monitoring: interim analysis	#21b	Description of any interim analyses and stopping guidelines, including who will have access to these interim results and make the final decision to terminate the trial	n/a
Harms	#22	Plans for collecting, assessing, reporting, and managing solicited and spontaneously reported adverse events and other unintended effects of trial interventions or trial conduct	n/a
Auditing	#23	Frequency and procedures for auditing trial conduct, if any, and whether the process will be independent from investigators and the sponsor	n/a
**Ethics and dissemination**			
Research ethics approval	#24	Plans for seeking research ethics committee / institutional review board (REC / IRB) approval	27
Protocol amendments	#25	Plans for communicating important protocol modifications (eg, changes to eligibility criteria, outcomes, analyses) to relevant parties (eg, investigators, REC / IRBs, trial participants, trial registries, journals, regulators)	27
Consent or assent	#26a	Who will obtain informed consent or assent from potential trial participants or authorised surrogates, and how (see Item 32)	13-14

For peer review only

		Reporting Item	Page Number
Consent or assent: ancillary studies	#26b	Additional consent provisions for collection and use of participant data and biological specimens in ancillary studies, if applicable	27
Confidentiality	#27	How personal information about potential and enrolled participants will be collected, shared, and maintained in order to protect confidentiality before, during, and after the trial	27
Declaration of interests	#28	Financial and other competing interests for principal investigators for the overall trial and each study site	28
Data access	#29	Statement of who will have access to the final trial dataset, and disclosure of contractual agreements that limit such access for investigators	27
Ancillary and post trial care	#30	Provisions, if any, for ancillary and post-trial care, and for compensation to those who suffer harm from trial participation	n/a
Dissemination policy: trial results	#31a	Plans for investigators and sponsor to communicate trial results to participants, healthcare professionals, the public, and other relevant groups (eg, via publication, reporting in results databases, or other data sharing arrangements), including any publication restrictions	27
Dissemination policy: authorship	#31b	Authorship eligibility guidelines and any intended use of professional writers	n/a
Dissemination policy: reproducible research	#31c	Plans, if any, for granting public access to the full protocol, participant-level dataset, and statistical code	27
**Appendices**			
Informed consent materials	#32	Model consent form and other related documentation given to participants and authorised surrogates	n/a
Biological specimens	#33	Plans for collection, laboratory evaluation, and storage of biological specimens for genetic or molecular analysis in the current trial and for future use in ancillary studies, if applicable	n/a

For peer review only

Table S2. World Health Organization Trial Registration Data Set

Data category	Information
Primary registry and trial identifying number	ISRCTN 86084138<br>https://doi.org/10.1186/ISRCTN86084138
Date of registration in primary registry	16 March, 2022
Secondary identifying numbers	n/a
Source(s) of monetary or material support	Bill & Melinda Gates Foundation; National Institute of Environmental Health Sciences
Primary sponsor	n/a
Secondary sponsor(s)	n/a
Contact for public queries	Joe Brown, PhD PE; joebrown@unc.edu
Contact for scientific queries	Joe Brown, PhD PE<br>Department of Environmental Sciences and Engineering<br>University of North Carolina at Chapel Hill
Public title	Health impacts on children born after an urban sanitation intervention in low-income neighborhoods in Mozambique: the Maputo Sanitation (MapSan) trial after five years
Scientific title	Long-term impact of an urban sanitation intervention on child health in low-income neighborhoods of Maputo city, Mozambique: a cross-sectional follow-up five years post-intervention in the Maputo Sanitation (MapSan) trial
Countries of recruitment	Mozambique
Health condition(s) or problem(s) studied	Shedding of enteric pathogens in stool and associated sequelae in children under 5 years of age
Intervention(s)	Intervention: existing onsite (non-sewered) pour-flush toilet to septic tank and soak pit for liquid effluent
	Control: comparable household clusters with unhygienic shared sanitation facilities in poor condition
Key inclusion and exclusion criteria	Ages eligible for study: 29 days – 60 months<br>Sexes eligible for study: both<br>Accepts healthy volunteers: yes
	Inclusion criteria: born into and residing in a compound enrolled in the five-year follow-up study; continuously resided in the study compound for the preceding 6 months, or since birth if under 6 months of age; parent or guardian who is able to understand and complete the written informed consent process and allow their child to participate
	Exclusion criteria: any caregiver-indicated medical condition or disability that precludes participation in the study
Study type	Observational, cross-sectional
	Allocation: non-randomized, unblinded
	Primary purpose: prevention

For peer review only - http://bmjopen.bmj.com/site/about/guidelines.xhtml

For peer review only

Data category	Information
	Trial setting: home
Date of first enrolment	28 March 2022
Target sample size	1100
Recruitment status	Recruiting
Primary outcome(s)	Prevalence of 22 bacterial, protozoan, and soil transmitted helminth (STH) enteric pathogens in child stool
Key secondary outcomes	Individual pathogen detection prevalence and gene copy density of 27 enteric pathogens (including viruses); mean height-for-age (HAZ), weight-for-age (WAZ), and weight-for-height (WHZ) z-scores; prevalence of stunting, underweight, and wasting; and the 7-day period-prevalence of caregiver-reported diarrhea.

For peer review only

BMJ Open

Long-term impacts of an urban sanitation intervention on enteric pathogens in children in Maputo city, Mozambique: study protocol for a cross-sectional follow-up to the Maputo Sanitation (MapSan) trial five years post-intervention

Journal:	*BMJ Open*
Manuscript ID	bmjopen-2022-067941.R1
Article Type:	Protocol
Date Submitted by the Author:	24-Jan-2023

Complete List of Authors:	Holcomb, David; The University of North Carolina at Chapel Hill Gillings School of Global Public Health, Environmental Sciences and Engineering<br>Monteiro, Vanessa; Centro de Investigação e Treino em Saúde da Polana Caniço, Instituto Nacional de Saúde<br>Capone, Drew; Indiana University, Department of Environmental and Occupational Health, School of Public Health<br>António, Virgílio; Centro de Investigação e Treino em Saúde da Polana Caniço, Instituto Nacional de Saúde<br>Chiluvane, Márcia; Centro de Investigação e Treino em Saúde da Polana Caniço, Instituto Nacional de Saúde<br>Cumbane, Victória; Centro de Investigação e Treino em Saúde da Polana Caniço, Instituto Nacional de Saúde<br>Ismael, Nália; Division of Biotechnology and Genetics, Instituto Nacional de Saúde<br>Knee, Jackie ; London School of Hygiene and Tropical Medicine Faculty of Infectious and Tropical Diseases<br>Kowalsky, Erin; The University of North Carolina at Chapel Hill Gillings School of Global Public Health, Department of Environmental Sciences and Engineering<br>Lai, Amanda; University of North Carolina at Chapel Hill Gillings School of Global Public Health, Environmental Science and Engineering<br>Linden, Yarrow; The University of North Carolina at Chapel Hill Gillings School of Global Public Health, Department of Environmental Sciences and Engineering<br>Mataveia, Elly; Centro de Investigação e Treino em Saúde da Polana Caniço, Instituto Nacional de Saúde<br>Nala, Rassul; Instituto Nacional de Saúde<br>Rao, Gouthami; The University of North Carolina at Chapel Hill Gillings School of Global Public Health, Department of Environmental Sciences and Engineering<br>Ribeiro, Jorge; Centro de Investigação e Treino em Saúde da Polana Caniço, Instituto Nacional de Saúde<br>Cumming, Oliver; London School of Hygiene and Tropical Medicine, Department of Disease Control<br>Viegas, Edna; Centro de Investigação e Treino em Saúde da Polana Caniço, Instituto Nacional de Saúde<br>Brown, Joe; University of North Carolina at Chapel Hill Gillings School of Global Public Health, Environmental Science and Engineering

**Primary Subject Heading**:	Global health
Secondary Subject Heading:	Epidemiology
Keywords:	Gastrointestinal infections < GASTROENTEROLOGY, Epidemiology < INFECTIOUS DISEASES, PUBLIC HEALTH

SCHOLARONE™
Manuscripts

TITLE

Long-term impacts of an urban sanitation intervention on enteric pathogens in children in Maputo city, Mozambique: study protocol for a cross-sectional follow-up to the Maputo Sanitation (MapSan) trial five years post-intervention

AUTHORS

David Holcomb[1,2], Vanessa Monteiro[3], Drew Capone[1,4], Virgílio António[5], Márcia Chiluvane,[3] Victória Cumbane[3], Nália Ismael[5], Jackie Knee[6], Erin Kowalsky[1], Amanda Lai[1], Yarrow Linden[1], Elly Mataveia[3], Rassul Nalá[7], Gouthami Rao[1], Jorge Ribeiro[3], Oliver Cumming[6], Edna Viegas[3], Joe Brown[1,*]

AFFILIATIONS

1. Department of Environmental Sciences and Engineering, Gillings School of Global Public Health, University of North Carolina at Chapel Hill, Chapel Hill, North Carolina, USA

2. Department of Epidemiology, Gillings School of Global Public Health, University of North Carolina at Chapel Hill, Chapel Hill, North Carolina, USA

3. Centro de Investigação e Treino em Saúde da Polana Caniço, Instituto Nacional de Saúde, Maputo, Mozambique

4. Department of Environmental and Occupational Health, School of Public Health, Indiana University, Bloomington, Indiana, USA

5. Division of Biotechnology and Genetics, Instituto Nacional de Saúde, Marracuene, Mozambique

For peer review only

For peer review only - http://bmjopen.bmj.com/site/about/guidelines.xhtml

6. Department of Disease Control, Faculty of Infectious and Tropical Diseases, London School of Hygiene and Tropical Medicine, London, UK

7. Division of Parasitology, Instituto Nacional de Saúde, Marracuene, Mozambique

CORRESPONDING AUTHOR

* Joe Brown; 135 Dauer Drive, Chapel Hill, NC, USA, 27599-7435; T: (919) 966-7892; E: joebrown@unc.edu

For peer review only

ABSTRACT

Introduction

We previously assessed the effect of an onsite sanitation intervention in informal neighborhoods of urban Maputo, Mozambique on enteric pathogen detection in children after two years of follow-up (Maputo Sanitation [MapSan] trial, clinicaltrials.gov: NCT02362932). We found significant reductions in *Shigella* and *Trichuris* prevalence but only among children born after the intervention was delivered. In this study, we assess the health impacts of the sanitation intervention after five years among children born into study households post-intervention.

Methods and analysis

We are conducting a cross-sectional household study of enteric pathogen detection in child stool and the environment at compounds (household clusters sharing sanitation and outdoor living space) that received the pour-flush toilet and septic tank intervention at least five years prior or meet the original criteria for trial control sites. We are enrolling at least 400 children (ages 29 days-60 months) in each treatment arm. Our primary outcome is the prevalence of 22 bacterial, protozoan, and soil transmitted helminth (STH) enteric pathogens in child stool using the pooled prevalence ratio across the outcome set to assess the overall intervention effect. Secondary outcomes include the individual pathogen detection prevalence and gene copy density of 27 enteric pathogens (including viruses); mean height-for-age (HAZ), weight-for-age (WAZ), and weight-for-height (WHZ) z-scores; prevalence of stunting, underweight, and wasting; and the 7-day period-prevalence of caregiver-reported diarrhea. All analyses are adjusted for pre-specified covariates and examined for effect measure modification by age. Environmental samples from study households and the public domain are assessed for pathogens and fecal indicators to explore environmental exposures and monitor disease transmission.

For peer review only - http://bmjopen.bmj.com/site/about/guidelines.xhtml

54 **Ethics and dissemination**

55 Study protocols have been reviewed and approved by human subjects review boards at the Ministry of Health, Republic of Mozambique and the University of North Carolina at Chapel Hill. De-identified study data will be deposited at https://osf.io/e7pvk/.

58 **Trial registration number**

59 ISRCTN86084138

For peer review only

For peer review only - http://bmjopen.bmj.com/site/about/guidelines.xhtml

KEYWORDS

Enteric infection; diarrheal disease; molecular pathogen detection; environmental microbiology; wastewater-based epidemiology

STRENGTHS AND LIMITATIONS OF THIS STUDY

- Long-term follow-up of an urban on-site sanitation intervention implemented at least five years prior so that all participants have been exposed to the treatment conditions for their entire lives.
- Primary study endpoint is molecular detection of multiple enteric pathogens in child stool, which unambiguously indicates previous exposure to specific sanitation-related pathogens.
- Primary outcome is the overall impact of the intervention on enteric pathogen exposure using a novel pooled estimate of the treatment effect across a pre-specified set of enteric pathogens.
- As an observational evaluation of an existing intervention, sample size is constrained by the number of eligible children residing at study sites and selection bias arising from differential enteric pathogen-related mortality may be present, particularly among older age groups.
- As a cross-sectional study, there is potential for confounding bias in our estimates of the intervention impacts on child health, particularly as the intervention itself may have influenced the desirability of the intervention sites and thus the socioeconomic status of their residents, which may be associated with reduced pathogen exposures.

For peer review only - http://bmjopen.bmj.com/site/about/guidelines.xhtml

INTRODUCTION

There is a high burden of childhood enteric infections associated with poor environmental conditions, with multiple enteric pathogens frequently detected in stool within the first year of life [1–3]. This is also the period with the highest incidence of diarrhea, which remains a leading cause of child mortality in low- and middle-income countries (LMIC) and has long been associated with stunted growth [4–6]. However, many diarrheal episodes are not attributable to infectious etiologies [3,7], while the etiology of attributable diarrhea varies widely by setting and only certain diarrheal pathogens are consistently implicated in reduced linear growth [2,8,9]. Far more prevalent is asymptomatic enteric pathogen shedding in stool [3,7,10–14], which may be more strongly associated with poor growth than diarrheal illness [2,15–17], potentially by contributing to intestinal inflammation, gut permeability, and nutrient malabsorption in a condition known as environmental enteric dysfunction (EED) [18–21]. In addition to the diverse negative impacts of stunting [22], specific adverse health outcomes associated with enteric infection and EED include delayed cognitive development [23–25] and reduced oral vaccine efficacy [26,27].

Water, sanitation, and hygiene (WASH) interventions aim to prevent diarrhea and improve child growth by interrupting fecal-oral pathogen transmission [28]. However, recent rigorous WASH intervention trials have demonstrated inconsistent and often limited impacts on child health [29–34,13]. Methodological limitations of such distal outcome measures notwithstanding [35,36], these findings are consistent with the interventions insufficiently interrupting environmental transmission and exposure to enteric pathogens [37]. Combined WASH interventions with high fidelity and adherence reduced stool-based detection of *Giardia*, hookworm, and possibly

For peer review only - http://bmjopen.bmj.com/site/about/guidelines.xhtml

Ascaris among 30 month-old children (but not younger children) and enteric viruses in rural Bangladesh [10,38,39], *Ascaris* in rural Kenya [40], and the number of co-detected parasites in rural Zimbabwe [11], but not bacterial pathogens or those most associated with stunting in any setting [2]. The sanitation-only intervention arm in rural Bangladesh reduced *Trichuris* prevalence in child stool [38] and may have reduced pathogenic *Escherichia coli* on child hands in more crowded households [41], while a sanitation intervention in rural Cambodia did not impact any pathogens measured in child stool [13]. Viewed collectively, a clear picture emerges of pervasive childhood polymicrobial exposures that were not meaningfully prevented by low-cost WASH interventions [42–44].

The preceding trials were all conducted in rural settings, but rapid urbanization has led to exceptional growth of densely populated informal settlements that lack basic services and present unique health challenges [45,46]. We have previously investigated whether an onsite sanitation intervention delivered in low-income neighborhoods of urban Maputo, Mozambique reduced enteric pathogen prevalence in child stool [47]. Although we similarly found no evidence of an effect on combined prevalence of pre-specified enteric pathogens in our primary analyses [34], additional evidence suggests that the sanitation intervention may have reduced exposures to enteric pathogens in the environment. The intervention was delivered with high fidelity and was widely used by intervention households after two years [48]. Exploratory sub-group analyses indicated that 24 months after the intervention, *Shigella* prevalence was halved (-51%; 95% confidence interval [CI]: -15%, -72%) and *Trichuris* prevalence reduced by three-quarters (-76%; 95% CI: -40%, -90%) among children born into the study compounds after the intervention was implemented, relative to children born into the control compounds after baseline [34].

For peer review only - http://bmjopen.bmj.com/site/about/guidelines.xhtml

Furthermore, overall pathogen prevalence, pathogen counts, *E. coli* gene copy density, and the individual prevalence of *Ascaris* and pathogenic *E. coli* were all significantly reduced in soil at the intervention latrine entrance [49,50], suggesting the intervention effectively contained human excreta. While animals have been implicated as major sources of pathogen exposure in other settings [51], only companion animals were frequently present at study households and a locally validated indicator of poultry fecal contamination (the most commonly observed non-companion animal type) was rarely detected in household environments [52]. Conversely, indicators of human fecal contamination were widespread [50] and the two pathogens most impacted by the intervention—*Shigella* and *Trichuris*—are considered anthroponotic [53], suggesting that human excreta was a primary source of enteric pathogens in this dense, urban setting and that human-associated pathogens were impacted by sustained exposure to the intervention.

Enteric pathogen carriage is highly age-dependent [8,12], but we were previously limited by the timing of our follow-up survey (24 months post-intervention) to children under two years of age when assessing impacts on those born into the intervention. Furthermore, it has been suggested that the typically short (1-2 year) follow-up periods often used in WASH evaluations may be inadequate for any longer-term benefits to manifest, potentially contributing to the limited observed effects [42]. Accordingly, we are conducting a cross-sectional follow-up study to better understand the long-term impacts of the sanitation intervention five years after it was implemented on child enteric pathogen exposures and shedding, diarrheal disease, and growth among children born into study households post-intervention.

For peer review only - http://bmjopen.bmj.com/site/about/guidelines.xhtml

STUDY OBJECTIVES

Primary objective

To measure the long-term effect of a shared, onsite urban sanitation intervention on the pooled prevalence of pre-specified enteric pathogen targets detected in children's stool at least 60 months post-intervention.

Hypotheses

H1. The risk of stool-based enteric pathogen detection among children 29 days-60 months old is reduced for children born into households that previously received the sanitation intervention.

H2. Children born into households that previously received the sanitation intervention experience delayed exposure to enteric pathogens relative to comparably aged children from non-intervention households, reflected in a greater reduction in the risk of enteric pathogen detection among younger age groups and attenuated reduction in risk among older children.

Secondary objectives

1. To measure the effect of a shared, onsite urban sanitation intervention program on the individual prevalence and density of pre-specified enteric pathogen targets detected in children's stool and environmental samples at least 60 months post-intervention.
2. To measure the effect of a shared, onsite urban sanitation intervention program on child growth and the prevalence of caregiver-reported diarrhea in children at least 60 months post intervention.

For peer review only - http://bmjopen.bmj.com/site/about/guidelines.xhtml

METHODS AND ANALYSIS

Study setting

This household-based study is being conducted in 11 *bairros* (neighborhoods) in the *Nhlamankulu* District and 5 *bairros* in the *KaMaxaquene* District of Maputo city, Mozambique. Households in these densely populated, low-income neighborhoods typically cluster into self-defined compounds delineated by a wall or boundary that share outdoor living space and sanitation facilities. Study activities with participating households are conducted primarily in the compound shared outdoor space by staff from the *Centro de Investigação e Treino em Saúde da Polana Caniço* (Polana Caniço Health Research and Training Center; CISPOC) in Maputo, Mozambique, from which the study is implemented and managed.

Study design

Urban sanitation intervention

The nongovernmental organization (NGO) Water and Sanitation for the Urban Poor (WSUP) implemented a sanitation intervention in 2015-2016 at approximately 300 compounds in informal urban neighborhoods of Maputo under a larger program led by the Water and Sanitation Programme (WSP) of the World Bank [47]. In these compounds, ranging between three and twenty-five households each, existing unhygienic shared latrines were replaced with pour-flush toilets and a septic tank with a soak-away pit for the liquid effluent. Two intervention designs employing the same sanitation technology were implemented, with approximately 50 communal sanitation blocks (CSBs) and 250 shared latrines (SLs) constructed across 11 neighborhoods that exhibited diversity across density and other key characteristics (susceptibility to flooding,

For peer review only - http://bmjopen.bmj.com/site/about/guidelines.xhtml

relative poverty, access to water and sanitation infrastructure) [12,52,54]. SLs served compounds with fewer than 21 residents and provided a single cabin with the intervention toilet, while CSBs provided an additional cabin for every 20 compound residents and other amenities including rainwater harvesting, municipal water connections and storage, and washing, bathing and laundry facilities [34,55]. WSUP also constructed facilities to the same specifications at other compounds in the neighborhoods prior to 2015 and continued to deliver these intervention designs in the years following 2016.

Original controlled before-and-after study

To evaluate the impact of the WSUP sanitation intervention on child health, we conducted the Maputo Sanitation (MapSan) trial, a controlled before-and-after study of enteric pathogen detection in child stool (clinicaltrials.gov: NCT02362932) [47]. WSUP selected intervention compounds using the following criteria: (1) residents shared sanitation in poor condition as determined by an engineer; (2) the compound was located in the pre-defined implementation neighborhoods; (3) there were no fewer than 12 residents; (4) residents were willing to contribute financially to construction costs; (5) sufficient space was available for construction of the new facility; (6) the compound was accessible for transportation of construction materials and tank-emptying activities; (7) the compound had access to a legal piped water supply; and (8) the groundwater level was deep enough for construction of a septic tank. Control compounds were selected according to criteria 1, 3, 4, and 7 from the 11 neighborhoods where the intervention was implemented and 5 additional neighborhoods with comparable characteristics [12].

For peer review only - http://bmjopen.bmj.com/site/about/guidelines.xhtml

The MapSan trial recruited an open cohort at three time-points: baseline (pre-intervention), 12 months post-intervention, and 24 months post-intervention; children were eligible to participate if older than 1 month at the time of enrollment and under 48 months at baseline. We enrolled intervention and control compounds concurrently to limit any differential effects of seasonality or other secular trends on the outcomes. We found no evidence that the sanitation intervention reduced the combined prevalence of 12 bacterial and protozoan enteric pathogens (the pre-specified primary outcome), the individual prevalence of any single bacterial, protozoan, STH, or viral pathogen, or the period prevalence of caregiver-reported diarrhea 12 and 24 months post-intervention [34]. However, exploratory analyses indicated the intervention may have been protective against bacterial and soil-transmitted helminth infections among the cohort who were born into the intervention and may have reduced the spread of some pathogens into latrine entrance soils.

Long-term cross-sectional follow-up study

We are revisiting MapSan trial compounds at least five years after the sanitation intervention to conduct a cross-sectional survey of children who were born after the intervention was implemented. Due to substantial population turnover [34], both intervention and control compounds are being identified using previously collected geolocation data and their locations confirmed by study staff during site identification visits. Any compounds identified during the course of these mapping efforts that have received the sanitation intervention at least five years prior (built by WSUP to the same specifications) or match the original enrollment criteria for MapSan control compounds are also invited to participate, irrespective of previous participation in the MapSan trial. As an existing intervention, neither staff nor participants can be blinded to

For peer review only - http://bmjopen.bmj.com/site/about/guidelines.xhtml

intervention status. We anticipate enrollment to continue for approximately one year and are again enrolling intervention and control compounds concurrently to limit any differential effects of seasonality and other secular trends across the enrollment period.

Patient and public involvement

Patients and/or the public were not involved in the design, conduct, reporting, or dissemination plans of this research.

Eligibility criteria

We attempt to enroll all eligible children in each compound that either previously participated in the MapSan trial, has previously received an intervention latrine, or meets the original enrollment criteria for MapSan control compounds [34]. Participant inclusion criteria include:

1. Child aged 29 days-60 months old
2. Born into and residing in a compound enrolled in the five-year follow-up study; in intervention compounds, the child must have been born following the delivery of the sanitation intervention
3. Has continuously resided in the study compound for the preceding 6 months, or since birth if under 6 months of age
4. Has a parent or guardian who is able to understand and complete the written informed consent process and allow their child to participate

Children are excluded if they have any caregiver-indicated medical condition or disability that precludes participation in the study.

For peer review only - http://bmjopen.bmj.com/site/about/guidelines.xhtml

Participant enrollment

Enrollment is conducted by trained study staff in either Portuguese or Changana, according to the respondent's preference, with written materials provided in Portuguese. We first obtain verbal consent from the compound leader to approach households in the compound for enrollment. We then seek written, informed consent to participate from the parent or guardian of each eligible child. Participation is entirely voluntary; guardians may decline their child's participation for any reason and can withdraw their child at any point. We began enrolling participants on 28 March 2022.

Household visits and procedures

After locating study compounds, the household of a participating child is visited twice, typically on consecutive days. On the first day, trained study staff conduct written consent procedures; administer compound, household, and child questionnaires; record child anthropometry measures; collect environmental samples; and request the child's caregiver to retain a sample of the child's stool. The household is revisited the following day to collect a stool sample from the child and complete environmental sample collection. If the stool sample is unavailable, the study staff coordinate an additional visit to retrieve the stool. In the event that 7 days pass since the initial visit without collection of a stool sample, a qualified nurse visits the child to obtain a rectal swab.

After collection of the stool sample, deworming is offered to all household members >1 year old who have not been dewormed in the past year, unless pregnant or breastfeeding. Deworming consultation and medication provision is conducted by Ministry of Health staff following the

For peer review only

For peer review only - http://bmjopen.bmj.com/site/about/guidelines.xhtml

national guidelines for deworming procedures. Deworming is offered in-kind to all household members and leverages the household interaction to provide an important public health service. Besides deworming, this study offers no direct benefit to children participating in this study. No incentives are provided to study participants, but we provide 50 meticais (approximately US$1) of mobile phone credit on the caregiver's preferred network for each child to compensate for the costs incurred in communicating with the study team to arrange household visits. Households from which we collect food and/or large-volume water samples are reimbursed 50 additional meticais to offset these expenses.

Environmental sample collection

We are sampling environmental compartments at a randomly selected subset of 100 intervention and 100 control compounds to represent compound- and household-level exposures [37]. At the entrance to the compound latrine we collect soil, flies, and a large volume air sample, as well as fecal sludge from the latrine or septic tank and any animal feces observed in the shared outdoor space [49,50,56,57]. One household is randomly selected among those households with children enrolled in the child health study, from which we collect swabs of flooring at the household entrance, flies in cooking area, prepared child's food, stored drinking water, and water from the household's primary source [52,58,59].

We are also collecting environmental samples from the public domain to conduct environmental surveillance of pathogens circulating in the community [56,60]; such samples are not linked to specific individuals in any way. Sample matrices are informed by the compartments on Maputo's Excreta Flow Diagram [61], including wastewater, surface and open drain water, soils in the

For peer review only

For peer review only - http://bmjopen.bmj.com/site/about/guidelines.xhtml

vicinity of solid and fecal waste disposal locations, and wastewater effluent from hospitals treating COVID-19 patients. We identify sample locations using satellite imagery in consultation with technicians responsible for sanitation and drainage in Maputo. Samples are collected with written permission from the municipal government on a weekly basis for matrices with a single available sampling location (e.g., wastewater treatment plant influent) and twice at all other locations (once in each the rainy and dry seasons) to prioritize the geographic distribution of sampling locations across Maputo city. We aim to collect approximately 100 samples of solids (soil, fecal sludge), 100 large-volume liquid samples (wastewater, surface water, and open drains) [62], and 100 passive samples of liquids [60].

Study outcomes

Enteric pathogen detection in stool

Stool-based molecular detection is performed for 27 enteric pathogens commonly implicated in both symptomatic and asymptomatic childhood infections globally, including those identified at the Global Enteric Multicenter Study (GEMS) study site in Manhiça, Mozambique [12,36,56,63]. Reverse-transcription quantitative polymerase chain reaction (RT-qPCR) is conducted by custom TaqMan Array Card (TAC; Thermo Fisher, Carlsbad, CA, USA) to simultaneously quantify genetic targets corresponding to 13 bacterial pathogens (*Aeromonas* spp.; *Campylobacter jejuni*/*coli*; *Clostridioides difficile*; *E. coli* O157; enteroaggregative *E. coli* (EAEC); enteropathogenic *E. coli* (EPEC); enterotoxigenic *E. coli* (ETEC); Shiga toxin-producing *E. coli* (STEC); enteroinvasive *E. coli* (EIEC)/*Shigella* spp.; *Helicobacter pylori*; *Plesiomonas shigelloides*; *Salmonella enterica*; *Vibrio cholerae*), 4 protozoan parasites (*Cryptosporidium* spp.; *Cyclospora cayetanensis*; *Entamoeba histolytica; Giardia* spp.), 5 soil

For peer review only - http://bmjopen.bmj.com/site/about/guidelines.xhtml

transmitted helminths (*Ascaris lumbricoides*; *Ancylostoma duodenale*; *Necator americanus*; *Strongyloides stercolaris*; *Trichuris trichiura*), and 5 enteric viruses (adenovirus 40/41; astrovirus; norovirus GI/GII; rotavirus; sapovirus) [64]. We also include the respiratory virus SARS-CoV-2 on the custom TAC to support surveillance through fecal waste streams [65,66].

Our primary outcome is the prevalence of a pre-specified subset of 22 enteric pathogens, including *Shigella* spp. and *T. trichiura*, the two pathogens most impacted at 24 months among children born after the intervention was delivered [34]. As in the original MapSan study, we exclude enteric viruses from the primary outcome due to greater potential for direct contact transmission, which is unlikely to be impacted by the intervention [34,47,67]. Because we anticipate a combined prevalence (detection of at least one pathogen in a given stool sample) near 100%, and the intervention could plausibly increase the prevalence of some pathogens while reducing others, we do not define a composite prevalence outcome [34,68]. Rather, we will estimate the effect of the intervention on the prevalence of each pathogen individually, along with a pooled estimate of the intervention effect across the outcome set to serve as a summary of intervention's impact on pathogen prevalence that is directly comparable to the individual effect estimates [69,70]. The pooled effect of a single intervention across a set of related outcomes may be thought of as the treatment effect on a *generic* enteric pathogen. The pathogens for which individual effects can be more precisely estimated (such as those with higher background prevalence) contribute more to the pooled estimate, which is recovered alongside the individual effects for each pathogen and shares the same scale, allowing this summary metric to be directly interpreted in the context of its components, augmenting rather than replacing the individual estimates. This stands in contrast to composite outcomes, which construct new metrics that are

For peer review only

For peer review only - http://bmjopen.bmj.com/site/about/guidelines.xhtml

related to, but fundamentally differ from, their component outcomes—the prevalence of *any* pathogen being conceptually distinct from the prevalence of a *particular* pathogen, for instance.

Secondary outcomes include the individual prevalence and the continuous gene copy density in stool of all 27 enteric pathogens assessed on TAC , including enteric viruses. Recognizing the potential challenges to interpretation due to pervasive exposure, persistent infection and rapid re-infection, and possible protective effects [71–74], we will also repeat the primary outcome analysis excluding *Giardia* from the outcome set.

Anthropometry

Child weight and recumbent length (child age < 24 months) or standing height (24-60 months) are assessed according to standard World Health Organization (WHO) protocols and transformed to age-adjusted z-scores using WHO reference populations to obtain height-for-age (HAZ), weight-for-age (WAZ), and weight-for-height (WHZ) z-scores [75,76]. Secondary outcomes include continuous HAZ, WAZ, and WHZ, as well as prevalence of binary growth outcomes stunting (HAZ < -2), underweight (WAZ < -2), and wasting (WHZ < -2) [13].

Caregiver reported illness

Caregiver surveys are administered to ascertain child diarrheal disease, defined as the passage of three or more loose or watery stools in a 24-hour period, or any bloody stool, in the past 7 days [77]. We also assess two caregiver-reported negative control outcomes for each child: the 7-day period-prevalence of bruises, scrapes, or abrasions and the 7-day period-prevalence of toothache [78]. We do not expect the intervention to impact either child bruising or toothache prevalence,

For peer review only

For peer review only - http://bmjopen.bmj.com/site/about/guidelines.xhtml

so significant differences in these outcomes by treatment arm would suggest possible bias in our caregiver-reported outcomes.

Pathogen detection in environmental matrices

Molecular detection of selected pathogens and fecal source tracking (FST) markers is performed for environmental samples from both the private (compound and household) and public domains using a second custom TAC [49,56,79,80]. A subset of the enteric pathogens assessed in stool is included on the environmental TAC (adenovirus 40/41; astrovirus; norovirus GI/GII; rotavirus; sapovirus; *Aeromonas* spp.; *C. jejuni*/*coli*; *C. difficile*; *E. coli* O157; EAEC; EPEC; ETEC; STEC; EIEC/*Shigella* spp.; *H. pylori*; *S. enterica*; *V. cholerae; Cryptosporidium* spp.; *E. histolytica; Giardia* spp.; *A. lumbricoides*; *A. duodenale*; *N. americanus*; *T. trichiura*), as well as select environmental and zoonotic pathogens (*Leptospira* spp; *Toxocara* spp.) [53,81,82], other human pathogens detectable in feces (SARS-CoV-2, Zika virus, HIV proviral DNA; *Plasmodium* spp.; *Mycobacterium tuberculosis*) [83–86], FST markers (human, poultry, and canine mitochondrial DNA; avian 16S rRNA) [87,88], and general bacterial and anthropogenic pollution/antimicrobial resistance markers (bacterial 16S rRNA; class 1 integron-integrase gene *intI1*) [89,90]. We also culture fecal indicator bacteria (total coliforms and *E. coli*) using the IDEXX Colilert-18 and Quanti-Tray 2000 system [88,91]. We conduct ongoing genomic surveillance of SARS-CoV-2 viral lineages in wastewater samples using amplicon-based Illumina next generation sequencing [92].

For peer review only - http://bmjopen.bmj.com/site/about/guidelines.xhtml

Statistical analysis

Enteric pathogen outcomes

We will use mixed effects models with varying slopes and intercepts to simultaneously estimate individual treatment effects for each pathogen and the weighted-average intervention effect across all pathogens included in the model. The modelling approach, including illustrative model specifications for both binary and continuous outcomes and interpretation of model parameters, is described in the pre-specified data analysis plan (https://osf.io/e7pvk/). Briefly, the observed value (detection or gene copy density) of each pathogen for every child will be included as a separate response in the model design matrix and the intercept, intervention effect slope, and the slopes of other covariates will all be allowed to vary by pathogen. The intercept will also vary by child and compound to account for repeated measures of multiple pathogens per child and multiple children per compound. Each set of pathogen-varying effects (e.g., the pathogen-specific intervention effect slopes) will be structured as arising from a population of parameters with shared mean and variance [93,94], where the population-level mean corresponds to the weighted-average expected effect across all pathogens and the population-level variance indicates the extent to which the effect may differ by pathogen. We will also estimate covariances between the sets of pathogen-varying effects to account for dependencies, for example if the effect of the intervention on a specific pathogen is greater when the background prevalence of that pathogen (represented by the pathogen-specific intercept) is also higher. By partially pooling information between pathogens, this approach provides adaptive shrinkage of the individual treatment effect estimates for each pathogen as well as an estimate of the generalized effect across pathogens [50,93]. Such partial pooling of effect estimates helps control the false discovery rate for individual outcomes, mitigating the need for *post hoc* multiple

For peer review only - http://bmjopen.bmj.com/site/about/guidelines.xhtml

comparison adjustments [69,70]. As a sensitivity analysis, we will also fit separate models for each pathogen, applying the Benjamini-Hochberg procedure to control the false discovery rate [95].

The mixed effects models will be specified as Bayesian hierarchical models with regularizing hyperpriors (see data analysis plan for discussion of prior distributions) and sampled using Markov chain Monte Carlo (MCMC) approaches [50,93,96]. For binary enteric pathogen detection outcomes, the canonical logistic link function will be used for computational stability and a Bayesian parametric g-formula algorithm will be applied to estimate marginal prevalence ratios and differences from the posterior predictive distribution [97]. Posterior predicted probabilities will be sampled for each pathogen assuming all participants received the intervention and again assuming none received the intervention, leaving all other covariate values unchanged, to obtain weighted averages over the distribution of confounders in the sample population, as in marginal standardization [98]. The posterior predicted prevalence ratio and prevalence difference distributions for each pathogen will be approximated by the ratio and difference, respectively, of the posterior predicted probability draws under the all-treated and none-treated scenarios. Differences in average gene copy density (scaled by pathogen-specific sample standard deviation to facilitate comparison across pathogens) will be estimated as the measure of effect using linear Bayesian hierarchical models for semicontinuous enteric pathogen quantity outcomes, with non-detects considered true zeros and censoring used to create a zero class (as in Tobit regression; refer to data analysis plan for implementation details) [96,99]. As a sensitivity analysis, we will also implement the two-stage parametric g-formula approach of Rogawski McQuade et al. to estimate differences in average quantity separately for each

For peer review only

For peer review only - http://bmjopen.bmj.com/site/about/guidelines.xhtml

pathogen, applying the Benjamini-Hochberg procedure to account for multiple comparisons [11]. Effect estimates will be summarized using the mean of the posterior predictive distribution to represent the expected effect size and the central 95% probability interval to describe the range of effect sizes compatible with the data (the 95% CI). Parameters with 95% CIs that exclude the null will be considered significant, although the magnitude and uncertainty of parameter estimates will also be considered holistically in evaluating evidence for clinically meaningful effects [100].

For peer review only

Growth and caregiver-reported outcomes

The effects of the intervention on mean HAZ, WAZ, and WHZ; the prevalence of stunting, underweight, and wasting; and the period-prevalence of caregiver-reported diarrhea and negative control outcomes (bruising, scrapes, and abrasions; toothache) will be analyzed separately as secondary outcomes using generalized estimating equations (GEE) and robust standard errors with exchangeable correlation structure and clustering by compound (the level at which the sanitation intervention was delivered) [13,34,101]. The estimated difference in age-adjusted z-scores by treatment assignment will be used as the measure of effect for continuous anthropometry outcomes. The prevalence ratio will be estimated by Poisson regression for binary growth status and caregiver-reported outcomes. We will not adjust for multiple comparisons [31,102].

Covariates and effect measure modification

As a cross-sectional study of an existing intervention, all analyses will be adjusted for a set of covariates selected *a priori* as potential confounders of the sanitation-enteric pathogen carriage

relationship [103]. The adjustment set will include child age and sex, caregiver's education, and household wealth index and additional covariates will be considered in exploratory adjusted analyses [10,11,34,104]; records missing covariate data will be excluded from primary analyses. Analyses will be repeated with missing data imputed by multiple imputation using chained equations (MICE) as a sensitivity analysis [34,105]. In addition to covariates, caregiver-reported outcomes and continuous growth outcomes (HAZ, WAZ, and WHZ) will used for imputation; derived binary growth statuses (stunting, underweight, and wasting) will be re-calculated for each observation after conducting imputation procedures. Missing pathogen outcomes will not be imputed, nor will pathogens be used to inform imputation of other variables. However, datasets containing imputed covariates will be joined to observed pathogen outcomes by unique child identifier for sensitivity analyses; the Bayesian hierarchical models will be fit to each imputed dataset and draws from the posterior predictive distributions for parameters and contrasts of interest will be concatenated to obtain pooled estimates across the multiple imputed datasets [106]. The specific enteric pathogens detected are expected to be strongly related to child age [8,34]. We will examine effect measure modification of the primary and secondary outcomes stratifying by age group (1-11 months, 12-23 months, and 24-60 months) [3,107].

Independently upgraded controls

We anticipate some of the control compounds may have independently upgraded their sanitation facilities to conditions comparable to the intervention. Control compounds with sanitation facilities observed to possess cleanable, intact hardscape slabs; pour-flush or water-sealed toilets; a functional ventilation pipe; and a fixed superstructure with sturdy walls and a secure door that ensure privacy during use are considered to have independently upgraded to conditions

For peer review only - http://bmjopen.bmj.com/site/about/guidelines.xhtml

comparable to the intervention [55]. Children living in control compounds with independently upgraded latrines are enrolled but will be excluded from the main analyses of the intervention effects. Two sets of subgroup analyses will instead be conducted that include all participants: one in which children in independently upgraded controls are considered as part of the control (non-intervention) arm, and again considered as part of the intervention arm. We will compare parameter estimates from the three sets of analyses to investigate whether the sanitation improvements independently available in the study communities are comparable to the full sanitation intervention package assessed in the MapSan trial in terms of child health impacts.

Eligible children residing in any compound that has received a WSUP intervention latrine will be considered part of the intervention arm in primary analyses. We record the current conditions of intervention facilities but will not exclude or otherwise adjust for either upgraded or degraded sanitation facilities in intervention compounds in order to evaluate the long-term impacts of the intervention following extended use.

Minimum detectable effect size

The number of participants will be constrained by the number of compounds in the study neighborhoods that have received the sanitation intervention or meet the eligibility requirements for MapSan control compounds, most of which were previously enrolled in the MapSan trial. At the 24 month follow-up, an average of 2.5 children per compound were enrolled from 408 compounds [34]. Compound-level intra-class correlation coefficients (ICCs) were generally less than 0.1 for individual pathogens, corresponding to cluster variances of ~0.05. We calculate the minimum detectable effect size (MDES) on individual pathogen prevalence with 80% power, 5%

For peer review only - http://bmjopen.bmj.com/site/about/guidelines.xhtml

significance level, and 0.05 compound cluster variance for a conservative scenario with 200 compounds per treatment arm and 2 children enrolled per compound (for 800 children total, 400 per arm), a moderate scenario of 220 compounds per treatment arm and 2.5 children enrolled per compound (550 children per arm, 1100 total), and a maximal scenario of 300 compounds per arm, 2.5 children per compound (750 children per arm, 1500 total) [108]. Across all scenarios, a minimum baseline (untreated) prevalence of 6-8% is required to reach 80% power for even the largest theoretical effect (nearly 100% reduction). The minimum detectable relative reduction in prevalence decreases (that is, smaller effect sizes are more readily detected) as pathogen prevalence increases towards 100% (Figure 1). The difference between the scenarios on the multiplicative scale is relatively minor, with the relative reduction MDES largely driven by pathogen baseline prevalence. A pathogen with baseline prevalence below 15% must have its prevalence halved ($PR < 0.5$) in order to attain 80% power, while a 25% reduction is detectable with 80% power for baseline prevalence of 34-46% under the maximal and conservative scenarios, respectively. We expect the simultaneous consideration of multiple pathogens to reduce the MDES for the primary outcome pooled intervention effect by effectively increasing the sample size. Because this pooled effect is dependent on the prevalence of each pathogen considered and the correlations between them, we will conduct simulation analyses to characterize plausible MDES ranges for the pooled primary outcome treatment effect [68,108].

For peer review only

For peer review only - http://bmjopen.bmj.com/site/about/guidelines.xhtml

LIMITATIONS

As an observational, cross-sectional evaluation of an existing intervention, this study faces a number of limitations that may impact the generalizability of our findings. By design, all of the study participants will have been born after the intervention was implemented in order to evaluate intervention impacts among individuals across a range of ages who have been exposed to intervention for their entire lives. However, there is potential for confounding bias in our estimates of the intervention impacts on child health, particularly concerning socioeconomic factors that may be associated with increased pathogen exposures. While non-randomized, the criteria for intervention and control compounds differed only by engineering considerations that we believe to be independent of the outcome. However, in the years since the intervention was implemented, the presence of the intervention itself may have influenced the desirability of the intervention sites and thus the socioeconomic status (SES) of their residents, particularly in light of the high population turnover previously observed after only 1-2 years. All analyses will be adjusted for a location-specific wealth index to account for potential differential SES between treatment arms [104], in addition to other pre-specified covariates associated with enteric pathogen exposure that may plausibly be related to treatment status, but the possibility of unmeasured and insufficiently controlled confounding remains.

Because childhood diarrhea is a leading cause of child mortality, selection bias arising from survivor effects may be present in our sample, particularly among older age groups [109]. However, loss to follow-up due to mortality was exceptionally rare in the previous assessment—far less common than emigration, which was not previously differential by treatment arm [34]. Our use of stool-based enteric pathogen detection as an objective primary outcome mitigates the

For peer review only - http://bmjopen.bmj.com/site/about/guidelines.xhtml

potential for measurement bias [36] and we are assessing multiple negative control outcomes to account for potential response bias in our secondary caregiver-reported outcomes [78]. As a long-term evaluation of an existing intervention, the potential for exposure measurement error is low—interventions that have degraded substantially may shift results towards the null, but in so doing would represent a valid assessment of intervention sustainability [110]. The potential for exposure misclassification among controls is higher, in that they may have independently upgraded to sanitation infrastructure comparable to the intervention. We are actively monitoring this possibility at all study sites; have pre-specified criteria for identifying sites achieving such conditions; and will conduct sensitivity analyses with these sites excluded, considered as controls, and considered as equivalent to the intervention to characterize the impact of any such potential exposure misclassification among controls.

A key challenge is identifying an appropriate set of outcomes against which to evaluate the intervention; in this regard, we preferred inclusivity given the pathogens likely to be observed in our study setting, at the risk of unduly shifting our eventual results towards the null. This challenge persists, regardless of study design, so long as the study aims include assessing the effect of some condition or intervention on exposure to, or infection by, multiple pathogens that share a common route of transmission [111].

CONTRIBUTION

Although observational, this study is unique in evaluating sanitation intervention effects at least five years after the intervention was implemented and up to five years after participants were borne into the intervention conditions. Previous studies have focused on WASH intervention

For peer review only

impacts up to 2-3 years after delivery [13,31–34], which may be insufficient time to realize impacts [42]. Furthermore, most previous studies have been conducted in rural settings, while we are investigating the long-term effects of an urban sanitation intervention that is broadly representative of the types of infrastructure improvements likely to be available in the rapidly growing urban informal settlements in coming years. Finally, we use stool-based detection of multiple enteric pathogens as an objective outcome and propose a novel pooled estimate of the treatment effect across a pre-specified outcome set to summarize the overall impact of the intervention on enteric pathogen exposure as the primary trial outcome.

For peer review only

For peer review only - http://bmjopen.bmj.com/site/about/guidelines.xhtml

ETHICS AND DISSEMINATION

This study was approved by Comité Nacional de Bioética para a Saúde, Ministério da Saúde de Moçambique (FWA#: 00003139, IRB00002657, 326/CNBS/21; approved: 15 June 2021) and University of North Carolina at Chapel Hill Ethics Committee (IRB#: 21-1119; approved 19 August 2021), and was prospectively registered with ISRCTN on 16 March 2022 (https://doi.org/10.1186/ISRCTN86084138). We pre-specified a statistical analysis plan that was deposited in a permanent online repository (https://osf.io/e7pvk/) prior to commencing enrollment. All protocol modification will be submitted to, and approved by, the respective ethics committees prior to implementation and promptly updated on the ISRCTN registry.

Written, informed consent is obtained from the parent(s)/guardian(s) of each participant. Identifiable information is maintained separately to protect confidentiality and linked to surveys, stool and environmental sample data by unique codes; the linking file to identifiable information will be destroyed upon completion of the study and the study data and remaining biospecimens retained for fully de-identified ancillary analyses. Results will be presented to key stakeholders in Mozambique, including local and national government officials, public utilities, and non-governmental organizations, and published in open access peer-reviewed journals. Upon publication of study results, the underlying individual participant data will be fully de-identified according to the Safe Harbor method and made freely available in the permanent online repository (https://osf.io/e7pvk/).

For peer review only - http://bmjopen.bmj.com/site/about/guidelines.xhtml

AUTHOR CONTRIBUTIONS

JB, EV, OC, RN, and JK designed the study, secured funding, and provided ongoing supervision. JB is also the corresponding author and guarantor. VM and DC drafted the initial study protocol and obtained ethical approvals. DH developed the statistical analysis plan, prepared the prospective trial registration and protocol manuscript, and is the first author. EK, GR, EM, JR, VA, AL, and YL developed and implemented the study procedures, which were overseen by VC, VM, MC, and NI. All authors contributed to and approved the final manuscript.

FUNDING

This work is funded by the Bill & Melinda Gates Foundation (OPP1137224) with additional support from a National Institute of Environmental Health Sciences training grant (T32ES007018). The funders had no role in the study design; data collection, analysis, and interpretation; or decision to publish.

COMPETING INTERESTS

The authors declare that they have no competing interests.

ACKNOWLEDGEMENTS

We thank all the participants, their families, and neighbors for graciously welcoming us into their communities, and our implementing partner, Water and Sanitation for the Urban Poor, for their continued support. We also gratefully acknowledge the hard work of the CISPOC survey team, including Jorge Binguane, Noémia Come, Anelsa Dunhe, Alice Fumo, Antônio Johane, Evelin

For peer review only - http://bmjopen.bmj.com/site/about/guidelines.xhtml

Matos, Eloisa Mula, Mariza Rachid, and Líria Sambo; and of Filipe Fazenda, Claúdia Machume, and Alfredo Muchanga in the CISPOC laboratory.

For peer review only

For peer review only - http://bmjopen.bmj.com/site/about/guidelines.xhtml

REFERENCES

1 Taniuchi M, Sobuz SU, Begum S, *et al.* Etiology of Diarrhea in Bangladeshi Infants in the First Year of Life Analyzed Using Molecular Methods. *Journal of Infectious Diseases* 2013;**208**:1794–802. doi:10.1093/infdis/jit507

2 Rogawski ET, Liu J, Platts-Mills JA, *et al.* Use of quantitative molecular diagnostic methods to investigate the effect of enteropathogen infections on linear growth in children in low-resource settings: longitudinal analysis of results from the MAL-ED cohort study. *The Lancet Global Health* 2018;**6**:e1319–28. doi:10.1016/S2214-109X(18)30351-6

3 Liu J, Platts-Mills JA, Juma J, *et al.* Use of quantitative molecular diagnostic methods to identify causes of diarrhoea in children: a reanalysis of the GEMS case-control study. *The Lancet* 2016;**388**:1291–301. doi:10.1016/S0140-6736(16)31529-X

4 Troeger CE, Khalil IA, Blacker BF, *et al.* Quantifying risks and interventions that have affected the burden of diarrhoea among children younger than 5 years: an analysis of the Global Burden of Disease Study 2017. *The Lancet Infectious Diseases* 2020;**20**:37–59. doi:10.1016/s1473-3099(19)30401-3

5 Black RE, Brown KH, Becker S. Effects of Diarrhea Associated with Specific Enteropathogens on the Growth of Children in Rural Bangladesh. *Pediatrics* 1984;**73**:799–805. doi:10.1542/peds.73.6.799

6 Benjamin-Chung J, Mertens A, Colford JM, *et al.* Early childhood linear growth faltering in low- and middle-income countries. medRxiv [preprint]. October 21, 2021 [cited 2022 Aug 30]. 2021. doi:10.1101/2020.06.09.20127001

7 Platts-Mills JA, Liu J, Rogawski ET, *et al.* Use of quantitative molecular diagnostic methods to assess the aetiology, burden, and clinical characteristics of diarrhoea in children in low-resource settings: a reanalysis of the MAL-ED cohort study. *The Lancet Global Health* 2018;**6**:e1309–18. doi:10.1016/S2214-109X(18)30349-8

8 Baker JM, Hasso-Agopsowicz M, Pitzer VE, *et al.* Association of enteropathogen detection with diarrhoea by age and high versus low child mortality settings: a systematic review and meta-analysis. *The Lancet Global Health* 2021;**9**:e1402–10. doi:10.1016/S2214-109X(21)00316-8

9 Schnee AE, Haque R, Taniuchi M, *et al.* Identification of Etiology-Specific Diarrhea Associated With Linear Growth Faltering in Bangladeshi Infants. *American Journal of Epidemiology* 2018;**187**:2210–8. doi:10.1093/aje/kwy106

10 Grembi JA, Lin A, Karim MA, *et al.* Effect of Water, Sanitation, Handwashing, and Nutrition Interventions on Enteropathogens in Children 14 Months Old: A Cluster-Randomized Controlled Trial in Rural Bangladesh. *The Journal of Infectious Diseases* Published Online First: August 2020. doi:10.1093/infdis/jiaa549

For peer review only - http://bmjopen.bmj.com/site/about/guidelines.xhtml

11 Rogawski McQuade ET, Platts-Mills JA, Gratz J, *et al.* Impact of Water Quality, Sanitation, Handwashing, and Nutritional Interventions on Enteric Infections in Rural Zimbabwe: The Sanitation Hygiene Infant Nutrition Efficacy (SHINE) Trial. *The Journal of Infectious Diseases* 2019;**221**:1379–86. doi:10.1093/infdis/jiz179

12 Knee J, Sumner T, Adriano Z, *et al.* Risk factors for childhood enteric infection in urban Maputo, Mozambique: A cross-sectional study. *PLOS Neglected Tropical Diseases* 2018;**12**:e0006956. doi:10.1371/journal.pntd.0006956

13 Lai AJ, Velez I, Ambikapathi R, *et al.* Independent and combined effects of nutrition and sanitation interventions on child growth and enteric pathogen carriage in rural Cambodia: a factorial cluster-randomised controlled trial. *medRxiv [preprint]* Published Online First: 2 September 2022. doi:10.1101/2021.05.21.21257546

14 Rego R, Watson S, Alam MAU, *et al.* A comparison of traditional diarrhoea measurement methods with microbiological and biochemical indicators: A cross-sectional observational study in the Cox's Bazar displaced persons camp. *eClinicalMedicine* 2021;**42**:101205. doi:10.1016/j.eclinm.2021.101205

15 Berendes D, Capone D, Knee J, *et al.* Associations between enteric pathogen carriage and height-for-age, weight-for-age and weight-for-height in children under 5 years old in urban Dhaka, Bangladesh. *Epidemiology and Infection* 2020;**148**:e39. doi:10.1017/s0950268820000369

16 Collard J-M, Andrianonimiadana L, Habib A, *et al.* High prevalence of small intestine bacteria overgrowth and asymptomatic carriage of enteric pathogens in stunted children in Antananarivo, Madagascar. *PLOS Neglected Tropical Diseases* 2022;**16**:1–22. doi:10.1371/journal.pntd.0009849

17 Garzón M, Pereira-da-Silva L, Seixas J, *et al.* Subclinical Enteric Parasitic Infections and Growth Faltering in Infants in São Tomé, Africa: A Birth Cohort Study. *International Journal of Environmental Research and Public Health* 2018;**15**. doi:10.3390/ijerph15040688

18 Prendergast AJ, Kelly P. Interactions between intestinal pathogens, enteropathy and malnutrition in developing countries. *Current opinion in infectious diseases* 2016;:1. doi:10.1097/QCO.0000000000000261

19 Prendergast AJ, Rukobo S, Chasekwa B, *et al.* Stunting Is Characterized by Chronic Inflammation in Zimbabwean Infants. *Plos One* 2014;**9**:11. doi:10.1371/journal.pone.0086928

20 George CM, Burrowes V, Perin J, *et al.* Enteric Infections in Young Children are Associated with Environmental Enteropathy and Impaired Growth. *Tropical Medicine & International Health* 2017;**12**:3218–21. doi:10.1111/tmi.13002

For peer review only - http://bmjopen.bmj.com/site/about/guidelines.xhtml

21 Kosek MN, Ahmed T, Bhutta Z, *et al.* Causal Pathways from Enteropathogens to Environmental Enteropathy: Findings from the MAL-ED Birth Cohort Study. *EBioMedicine* 2017;**18**:109–17. doi:10.1016/j.ebiom.2017.02.024

22 Hoddinott J, Behrman JR, Maluccio JA, *et al.* Adult consequences of growth failure in early childhood. *American Journal of Clinical Nutrition* 2013;**98**:1170–8. doi:10.3945/ajcn.113.064584

23 MAL-ED Network Investigators. Early childhood cognitive development is affected by interactions among illness, diet, enteropathogens and the home environment: findings from the MAL-ED birth cohort study. *BMJ Global Health* 2018;**3**:e000752. doi:10.1136/bmjgh-2018-000752

24 Oriá RB, Murray-Kolb LE, Scharf RJ, *et al.* Early-life enteric infections: relation between chronic systemic inflammation and poor cognition in children. *Nutrition Reviews* 2016;**74**:374–86. doi:10.1093/nutrit/nuw008

25 Donowitz JR, Drew J, Taniuchi M, *et al.* Diarrheal Pathogens Associated With Growth and Neurodevelopment. *Clinical Infectious Diseases* 2021;**73**:e683–91. doi:10.1093/cid/ciaa1938

26 Parker EP, Ramani S, Lopman BA, *et al.* Causes of impaired oral vaccine efficacy in developing countries. *Future Microbiology* 2018;**13**:97–118. doi:10.2217/fmb-2017-0128

27 Taniuchi M, Platts-Mills JA, Begum S, *et al.* Impact of enterovirus and other enteric pathogens on oral polio and rotavirus vaccine performance in Bangladeshi infants. *Vaccine* 2016;**34**:3068–75. doi:10.1016/j.vaccine.2016.04.080

28 Cumming O, Cairncross S. Can water, sanitation and hygiene help eliminate stunting? Current evidence and policy implications. *Maternal & Child Nutrition* 2016;**12**:91–105. doi:10.1111/mcn.12258

29 Wolf J, Hubbard S, Brauer M, *et al.* Effectiveness of interventions to improve drinking water, sanitation, and handwashing with soap on risk of diarrhoeal disease in children in low-income and middle-income settings: a systematic review and meta-analysis. *The Lancet* 2022;**400**:48–59. doi:10.1016/s0140-6736(22)00937-0

30 Contreras JD, Eisenberg JNS. Does Basic Sanitation Prevent Diarrhea? Contextualizing Recent Intervention Trials through a Historical Lens. *International Journal of Environmental Research and Public Health* 2020;**17**. doi:10.3390/ijerph17010230

31 Luby SP, Rahman M, Arnold BF, *et al.* Effects of water quality, sanitation, handwashing, and nutritional interventions on diarrhoea and child growth in rural Bangladesh: a cluster randomised controlled trial. *The Lancet Global Health* 2018;**6**:e302–15. doi:10.1016/s2214-109x(17)30490-4

32 Null C, Stewart CP, Pickering AJ, *et al.* Effects of water quality, sanitation, handwashing, and nutritional interventions on diarrhoea and child growth in rural Kenya: a cluster-

For peer review only

For peer review only - http://bmjopen.bmj.com/site/about/guidelines.xhtml

randomised controlled trial. *The Lancet Global Health* 2018;**6**:e316–29. doi:10.1016/s2214-109x(18)30005-6

33 Humphrey JH, Mbuya MNN, Ntozini R, *et al.* Independent and combined effects of improved water, sanitation, and hygiene, and improved complementary feeding, on child stunting and anaemia in rural Zimbabwe: a cluster-randomised trial. *The Lancet Global Health* 2019;**7**:e132–47. doi:10.1016/s2214-109x(18)30374-7

34 Knee J, Sumner T, Adriano Z, *et al.* Effects of an urban sanitation intervention on childhood enteric infection and diarrhea in Maputo, Mozambique: a controlled before-and-after trial. *eLife* 2021;**10**:e62278. doi:10.7554/elife.62278

35 Watson SI, Rego RTT, Hofer T, *et al.* Evaluations of water, sanitation and hygiene interventions should not use diarrhoea as (primary) outcome. *BMJ Global Health* 2022;**7**. doi:10.1136/bmjgh-2022-008521

36 Brown J, Cumming O. Stool-Based Pathogen Detection Offers Advantages as an Outcome Measure for Water, Sanitation, and Hygiene Trials. *The American Journal of Tropical Medicine and Hygiene* 2020;**102**:260–1. doi:10.4269/ajtmh.19-0639

37 Goddard F, Ban R, Barr DB, *et al.* Measuring environmental exposure to enteric pathogens in low-income settings: review and recommendations of an interdisciplinary working group. *Environ Sci Technol* 2020;:acs.est.0c02421. doi:10.1021/acs.est.0c02421

38 Ercumen A, Benjamin-Chung J, Arnold BF, *et al.* Effects of water, sanitation, handwashing and nutritional interventions on soil-transmitted helminth infections in young children: A cluster-randomized controlled trial in rural Bangladesh. *PLoS Neglected Tropical Diseases* 2019;**13**:1–24. doi:10.1371/journal.pntd.0007323

39 Lin A, Ercumen A, Benjamin-Chung J, *et al.* Effects of water, sanitation, handwashing, and nutritional interventions on child enteric protozoan infections in rural Bangladesh: A cluster-randomized controlled trial. *Clinical Infectious Diseases* 2018;**67**:1515–22. doi:10.1093/cid/ciy320

40 Pickering AJ, Njenga SM, Steinbaum L, *et al.* Effects of single and integrated water, sanitation, handwashing, and nutrition interventions on child soil-transmitted helminth and Giardia infections: A cluster-randomized controlled trial in rural Kenya. *PLOS Medicine* 2019;**16**:e1002841. doi:10.1371/journal.pmed.1002841

41 Fuhrmeister ER, Ercumen A, Pickering AJ, *et al.* Effect of sanitation improvements on pathogens and microbial source tracking markers in the rural Bangladeshi household environment. *Environ Sci Technol* 2020;:acs.est.9b04835. doi:10.1021/acs.est.9b04835

42 Pickering AJ, Null C, Winch PJ, *et al.* The WASH Benefits and SHINE trials: interpretation of WASH intervention effects on linear growth and diarrhoea. *The Lancet Global Health* 2019;**7**:e1139–46. doi:10.1016/s2214-109x(19)30268-2

For peer review only - http://bmjopen.bmj.com/site/about/guidelines.xhtml

43 Amebelu A, Ban R, Bhagwan J, *et al.* The Lancet Commission on water, sanitation and hygiene, and health. *The Lancet* 2021;**398**:1469–70. doi:10.1016/S0140-6736(21)02005-5

44 Budge S, Ambelu A, Bartram J, *et al.* Environmental sanitation and the evolution of water, sanitation and hygiene. *Bull World Health Organ* 2022;**100**:286–8. doi:10.2471/BLT.21.287137

45 Ezeh A, Oyebode O, Satterthwaite D, *et al.* The history, geography, and sociology of slums and the health problems of people who live in slums. *The Lancet* 2017;**389**:547–58. doi:10.1016/S0140-6736(16)31650-6

46 Sinharoy SS, Pittluck R, Clasen T. Review of drivers and barriers of water and sanitation policies for urban informal settlements in low-income and middle-income countries. *Utilities Policy* 2019;**60**:100957. doi:10.1016/j.jup.2019.100957

47 Brown J, Cumming O, Bartram J, *et al.* A controlled, before-and-after trial of an urban sanitation intervention to reduce enteric infections in children: research protocol for the Maputo Sanitation (MapSan) study, Mozambique. *BMJ Open* 2015;**5**:e008215–e008215. doi:10.1136/bmjopen-2015-008215

48 Bick S, Buxton H, Chase RP, *et al.* Using path analysis to test theory of change: a quantitative process evaluation of the MapSan trial. *BMC Public Health* 2021;**21**:1411. doi:10.1186/s12889-021-11364-w

49 Capone D, Berendes D, Cumming O, *et al.* Impact of an Urban Sanitation Intervention on Enteric Pathogen Detection in Soils. *Environ Sci Technol* 2021;**55**:9989–10000. doi:10.1021/acs.est.1c02168

50 Holcomb DA, Knee J, Capone D, *et al.* Impacts of an Urban Sanitation Intervention on Fecal Indicators and the Prevalence of Human Fecal Contamination in Mozambique. *Environ Sci Technol* 2021;**55**:11667–79. doi:10.1021/acs.est.1c01538

51 Fuhrmeister ER, Ercumen A, Pickering AJ, *et al.* Predictors of Enteric Pathogens in the Domestic Environment from Human and Animal Sources in Rural Bangladesh. *Environmental Science & Technology* 2019;**53**:10023–33. doi:10.1021/acs.est.8b07192

52 Holcomb DA, Knee J, Sumner T, *et al.* Human fecal contamination of water, soil, and surfaces in households sharing poor-quality sanitation facilities in Maputo, Mozambique. *International Journal of Hygiene and Environmental Health* 2020;**226**:113496. doi:10.1016/j.ijheh.2020.113496

53 Delahoy MJ, Wodnik B, McAliley L, *et al.* Pathogens Transmitted in Animal Feces in Low- and Middle-Income Countries. *International Journal of Hygiene and Environmental Health* 2018. doi:10.1016/j.ijheh.2018.03.005

54 Capone D, Adriano Z, Berendes D, *et al.* A localized sanitation status index as a proxy for fecal contamination in urban Maputo, Mozambique. *PLOS ONE* 2019;**14**:e0224333. doi:10.1371/journal.pone.0224333

For peer review only

For peer review only - http://bmjopen.bmj.com/site/about/guidelines.xhtml

55 Shiras T, Cumming O, Brown J, *et al.* Shared latrines in Maputo, Mozambique: exploring emotional well-being and psychosocial stress. *BMC International Health and Human Rights* 2018;**18**:30. doi:10.1186/s12914-018-0169-z

56 Capone D, Berendes D, Cumming O, *et al.* Analysis of Fecal Sludges Reveals Common Enteric Pathogens in Urban Maputo, Mozambique. *Environ Sci Technol Lett* 2020;:acs.estlett.0c00610. doi:10.1021/acs.estlett.0c00610

57 Ginn O, Rocha-Melogno L, Bivins A, *et al.* Detection and Quantification of Enteric Pathogens in Aerosols Near Open Wastewater Canals in Cities with Poor Sanitation. *Environ Sci Technol* 2021;**55**:14758–71. doi:10.1021/acs.est.1c05060

58 Bick S, Perieres L, D'Mello-Guyett L, *et al.* Risk factors for child food contamination in low-income neighbourhoods of Maputo, Mozambique: An exploratory, cross-sectional study. *Maternal & Child Nutrition* 2020;:e12991. doi:10.1111/mcn.12991

59 Exum NG, Olórtegui MP, Yori PP, *et al.* Floors and Toilets: Association of Floors and Sanitation Practices with Fecal Contamination in Peruvian Amazon Peri-Urban Households. *Environmental Science and Technology* 2016;**50**:7373–81. doi:10.1021/acs.est.6b01283

60 Schang C, Crosbie ND, Nolan M, *et al.* Passive Sampling of SARS-CoV-2 for Wastewater Surveillance. *Environ Sci Technol* 2021;**55**:10432–41. doi:10.1021/acs.est.1c01530

61 Peal A, Evans B, Blackett I, *et al.* Fecal sludge management (FSM): analytical tools for assessing FSM in cities. *Journal of Water, Sanitation and Hygiene for Development* 2014;**4**:371. doi:10.2166/washdev.2014.139

62 Zhou NA, Fagnant-Sperati CS, Komen E, *et al.* Feasibility of the Bag-Mediated Filtration System for Environmental Surveillance of Poliovirus in Kenya. *Food and Environmental Virology* 2020;**12**:35–47. doi:10.1007/s12560-019-09412-1

63 Kotloff KL, Nataro JP, Blackwelder WC, *et al.* Burden and aetiology of diarrhoeal disease in infants and young children in developing countries (the Global Enteric Multicenter Study, GEMS): A prospective, case-control study. *The Lancet* 2013;**382**:209–22. doi:10.1016/s0140-6736(13)60844-2

64 Liu J, Gratz J, Amour C, *et al.* Optimization of Quantitative PCR Methods for Enteropathogen Detection. *PLOS ONE* 2016;**11**:e0158199. doi:10.1371/journal.pone.0158199

65 Natarajan A, Zlitni S, Brooks EF, *et al.* Gastrointestinal symptoms and fecal shedding of SARS-CoV-2 RNA suggest prolonged gastrointestinal infection. *Med* 2022;**3**:371-387.e9. doi:10.1016/j.medj.2022.04.001

66 Bivins A, North D, Ahmad A, *et al.* Wastewater-Based Epidemiology: Global Collaborative to Maximize Contributions in the Fight Against COVID-19. *Environ Sci Technol* 2020;:acs.est.0c02388. doi:10.1021/acs.est.0c02388

For peer review only

For peer review only - http://bmjopen.bmj.com/site/about/guidelines.xhtml

67 Wikswo ME, Kambhampati A, Shioda K, *et al.* Outbreaks of Acute Gastroenteritis Transmitted by Person-to-Person Contact, Environmental Contamination, and Unknown Modes of Transmission — United States, 2009–2013. *MMWR Surveill Summ* 2015;**64**:1–16. doi:10.15585/mmwr.ss6412a1

68 Vickerstaff V, Ambler G, Omar RZ. A comparison of methods for analysing multiple outcome measures in randomised controlled trials using a simulation study. *Biometrical Journal* 2021;**63**:599–615. doi:10.1002/bimj.201900040

69 Gelman A, Hill J, Yajima M. Why We (Usually) Don't Have to Worry About Multiple Comparisons. *Journal of Research on Educational Effectiveness* 2012;**5**:189–211. doi:10.1080/19345747.2011.618213

70 Mitchell S, Gelman A, Ross R, *et al.* The Millennium Villages Project: a retrospective, observational, endline evaluation. *The Lancet Global Health* 2018;**6**:e500–13. doi:10.1016/s2214-109x(18)30065-2

71 Donowitz JR, Alam M, Kabir M, *et al.* A Prospective Longitudinal Cohort to Investigate the Effects of Early Life Giardiasis on Growth and All Cause Diarrhea. *Clinical Infectious Diseases* 2016;**63**:792–7. doi:10.1093/cid/ciw391

72 Bartelt LA, Platts-Mills JA. Giardia: a pathogen or commensal for children in high-prevalence settings? *Current Opinion in Infectious Diseases* 2016;**29**:502–7. doi:10.1097/qco.0000000000000293

73 Rogawski ET, Bartelt LA, Platts-Mills JA, *et al.* Determinants and Impact of Giardia Infection in the First 2 Years of Life in the MAL-ED Birth Cohort. *Journal of the Pediatric Infectious Diseases Society* 2017;**6**:153–60. doi:10.1093/jpids/piw082

74 Messa A, Köster PC, Garrine M, *et al.* Molecular diversity of Giardia duodenalis in children under 5 years from the Manhiça district, Southern Mozambique enrolled in a matched case-control study on the aetiology of diarrhoea. *PLoS Negl Trop Dis* 2021;**15**:e0008987. doi:10.1371/journal.pntd.0008987

75 de Onis M, Garza C, Victora CG, *et al.* The WHO Multicentre Growth Reference Study: Planning, Study Design, and Methodology. *Food Nutr Bull* 2004;**25**:S15–26. doi:10.1177/15648265040251S104

76 WHO. *Length/height-for-age, weight-for-age, weight-for-length, weight-for-height and body mass index-for-age: Methods and development*. Geneva: : World Health Organization 2006.

77 Arnold BF, Galiani S, Ram PK, *et al.* Optimal Recall Period for Caregiver-reported Illness in Risk Factor and Intervention Studies: A Multicountry Study. *American Journal of Epidemiology* 2013;**177**:361–70. doi:10.1093/aje/kws281

For peer review only

For peer review only - http://bmjopen.bmj.com/site/about/guidelines.xhtml

78 Arnold BF, Ercumen A, Benjamin-Chung J, *et al.* Brief Report: Negative Controls to Detect Selection Bias and Measurement Bias in Epidemiologic Studies. *Epidemiology* 2016;**27**:637–41. doi:10.1097/EDE.0000000000000504

79 Lappan R, Henry R, Chown SL, *et al.* Monitoring of diverse enteric pathogens across environmental and host reservoirs with TaqMan array cards and standard qPCR: a methodological comparison study. *The Lancet Planetary Health* 2021;**5**:e297–308. doi:10.1016/S2542-5196(21)00051-6

80 Baker KK, Senesac R, Sewell D, *et al.* Fecal Fingerprints of Enteric Pathogen Contamination in Public Environments of Kisumu, Kenya, Associated with Human Sanitation Conditions and Domestic Animals. *Environmental Science & Technology* 2018;**52**:10263–74. doi:10.1021/acs.est.8b01528

81 Rostami A, Ma G, Wang T, *et al.* Human toxocariasis – A look at a neglected disease through an epidemiological ‘prism.’ *Infection, Genetics and Evolution* 2019;**74**:104002. doi:10.1016/j.meegid.2019.104002

82 Schneider AG, Casanovas-Massana A, Hacker KP, *et al.* Quantification of pathogenic Leptospira in the soils of a Brazilian urban slum. *PLOS Neglected Tropical Diseases* 2018;**12**:1–15. doi:10.1371/journal.pntd.0006415

83 Muirhead A, Zhu K, Brown J, *et al.* Zika Virus RNA Persistence in Sewage. *Environ Sci Technol Lett* 2020;**7**:659–64. doi:10.1021/acs.estlett.0c00535

84 Chakrabarti AK, Caruso L, Ding M, *et al.* Detection of HIV-1 RNA/DNA and CD4 mRNA in feces and urine from chronic HIV-1 infected subjects with and without anti-retroviral therapy. *AIDS Res Ther* 2009;**6**:20. doi:10.1186/1742-6405-6-20

85 Jirků M, Pomajbíková K, Petrželková KJ, *et al.* Detection of *Plasmodium* spp. in Human Feces. *Emerg Infect Dis* 2012;**18**. doi:10.3201/eid1804.110984

86 Sultana S, Ansar A, Saif-Ur-Rahman KM. Stool specimen for diagnosis of pulmonary tuberculosis in adults: protocol for a systematic review and meta-analysis. *BMJ Open* 2021;**11**:e052212. doi:10.1136/bmjopen-2021-052212

87 Zhu K, Suttner B, Pickering A, *et al.* A novel droplet digital PCR human mtDNA assay for fecal source tracking. *Water Research* 2020;:116085. doi:10.1016/j.watres.2020.116085

88 Holcomb DA, Stewart JR. Microbial Indicators of Fecal Pollution: Recent Progress and Challenges in Assessing Water Quality. *Curr Envir Health Rpt* 2020;**7**:311–24. doi:10.1007/s40572-020-00278-1

89 Gillings MR, Gaze WH, Pruden A, *et al.* Using the class 1 integron-integrase gene as a proxy for anthropogenic pollution. *The ISME Journal* 2015;**9**:1269–79. doi:10.1038/ismej.2014.226

For peer review only - http://bmjopen.bmj.com/site/about/guidelines.xhtml

90 Keenum I, Liguori K, Calarco J, *et al.* A framework for standardized qPCR-targets and protocols for quantifying antibiotic resistance in surface water, recycled water and wastewater. *null* 2022;:1–25. doi:10.1080/10643389.2021.2024739

91 Julian TR, Islam MA, Pickering AJ, *et al.* Genotypic and Phenotypic Characterization of Escherichia coli Isolates from Feces, Hands, and Soils in Rural Bangladesh via the Colilert Quanti-Tray System. *Appl Environ Microbiol* 2015;**81**:1735–43. doi:10.1128/AEM.03214-14

92 Karthikeyan S, Levy JI, De Hoff P, *et al.* Wastewater sequencing reveals early cryptic SARS-CoV-2 variant transmission. *Nature* Published Online First: 7 July 2022. doi:10.1038/s41586-022-05049-6

93 McElreath R. *Statistical Rethinking: a Bayesian course with examples in R and Stan*. 2nd ed. Boca Raton: : Taylor and Francis, CRC Press 2020.

94 Bürkner P-C. brms: An R Package for Bayesian Multilevel Models Using Stan. *Journal of Statistical Software* 2017;**80**. doi:10.18637/jss.v080.i01

95 Benjamini Y, Hochberg Y. Controlling the False Discovery Rate: A Practical and Powerful Approach to Multiple Testing. *Journal of the Royal Statistical Society: Series B (Methodological)* 1995;**57**:289–300. doi:10.1111/j.2517-6161.1995.tb02031.x

96 Holcomb D, Capone C, Cumbane V, *et al.* Data Analysis Plan: Long-term impact of an urban sanitation intervention on child health in low-income neighborhoods of Maputo city, Mozambique: a cross-sectional follow-up five years post-intervention in the Maputo Sanitation (MapSan) trial. Version 4 (revised 2023-01-20). Published Online First: 20 January 2023.https://osf.io/e7pvk/

97 Keil AP, Daza EJ, Engel SM, *et al.* A Bayesian approach to the g-formula. *Statistical Methods in Medical Research* 2018;**27**:3183–204. doi:10.1177/0962280217694665

98 Muller CJ, MacLehose RF. Estimating predicted probabilities from logistic regression: different methods correspond to different target populations. *International Journal of Epidemiology* 2014;**43**:962–70. doi:10.1093/ije/dyu029

99 Clark JS, Nemergut D, Seyednasrollah B, *et al.* Generalized joint attribute modeling for biodiversity analysis: median-zero, multivariate, multifarious data. *Ecological Monographs* 2017;**87**:34–56. doi:10.1002/ecm.1241

100 Rafi Z, Greenland S. Semantic and cognitive tools to aid statistical science: replace confidence and significance by compatibility and surprise. *BMC Medical Research Methodology* 2020;**20**:244. doi:10.1186/s12874-020-01105-9

101 Bottomley C, Kirby MJ, Lindsay SW, *et al.* Can the buck always be passed to the highest level of clustering? *BMC Medical Research Methodology* 2016;**16**:29. doi:10.1186/s12874-016-0127-1

For peer review only - http://bmjopen.bmj.com/site/about/guidelines.xhtml

102 Rothman KJ. No Adjustments Are Needed for Multiple Comparisons. *Epidemiology* 1990;**1**. doi:10.1097/00001648-199001000-00010

103 VanderWeele TJ. Principles of confounder selection. *Eur J Epidemiol* 2019;**34**:211–9. doi:10.1007/s10654-019-00494-6

104 Schreiner M. Simple Poverty Scorecard Poverty-Assessment Tool Mozambique. Microfinance Risk Management, LLC 2017. https://www.simplepovertyscorecard.com/MOZ_2014_ENG.pdf

105 Buuren S van, Groothuis-Oudshoorn K. mice: Multivariate Imputation by Chained Equations in R. *Journal of Statistical Software* 2011;**45**. doi:10.18637/jss.v045.i03

106 Zhou X, Reiter JP. A Note on Bayesian Inference After Multiple Imputation. *The American Statistician* 2010;**64**:159–63. doi:10.1198/tast.2010.09109

107 VanderWeele TJ, Knol MJ. A Tutorial on Interaction. *Epidemiologic Methods* 2014;**3**:33–72. doi:10.1515/em-2013-0005

108 Arnold BF, Hogan DR, Colford JMJ, *et al.* Simulation methods to estimate design power: an overview for applied research. *BMC Medical Research Methodology* 2011;**11**:94. doi:10.1186/1471-2288-11-94

109 Hernán MA, Hernández-Díaz S, Robins JM. A Structural Approach to Selection Bias. *Epidemiology* 2004;**15**:615–25. doi:10.1097/01.ede.0000135174.63482.43

110 Goddard FGB, Chang HH, Clasen TF, *et al.* Exposure measurement error and the characterization of child exposure to fecal contamination in drinking water. *npj Clean Water* 2020;**3**:19. doi:10.1038/s41545-020-0063-9

111 Lash TL, Fox MP, Maclehose RF, *et al.* Good practices for quantitative bias analysis. *International Journal of Epidemiology* 2014;**43**:1969–85. doi:10.1093/ije/dyu149

For peer review only

For peer review only - http://bmjopen.bmj.com/site/about/guidelines.xhtml

FIGURE CAPTION

Figure 1. Range of minimum detectable effect sizes (shaded region) for the percent reduction in pathogen prevalence with 80% power, 5% significance level, and 0.05 cluster variance across three sample size scenarios. The upper edge of the shaded area represents a conservative scenario with 800 total participants (2 per compound, 200 compounds per arm) while the lower edge corresponds to a maximal scenario with 1500 total participants (2.5 per compound, 300 compounds per arm). A moderate scenario with 1100 total participants (2.5 per compound, 220 compounds per arm) is represented by the black curve within the shaded area. The vertical lines show the prevalence of a subset of pathogens assessed in control compound children during the 24-month follow-up in the original MapSan trial. Line color indicates the specific pathogen and line pattern reflects the pathogen class.

For peer review only

For peer review only - http://bmjopen.bmj.com/site/about/guidelines.xhtml

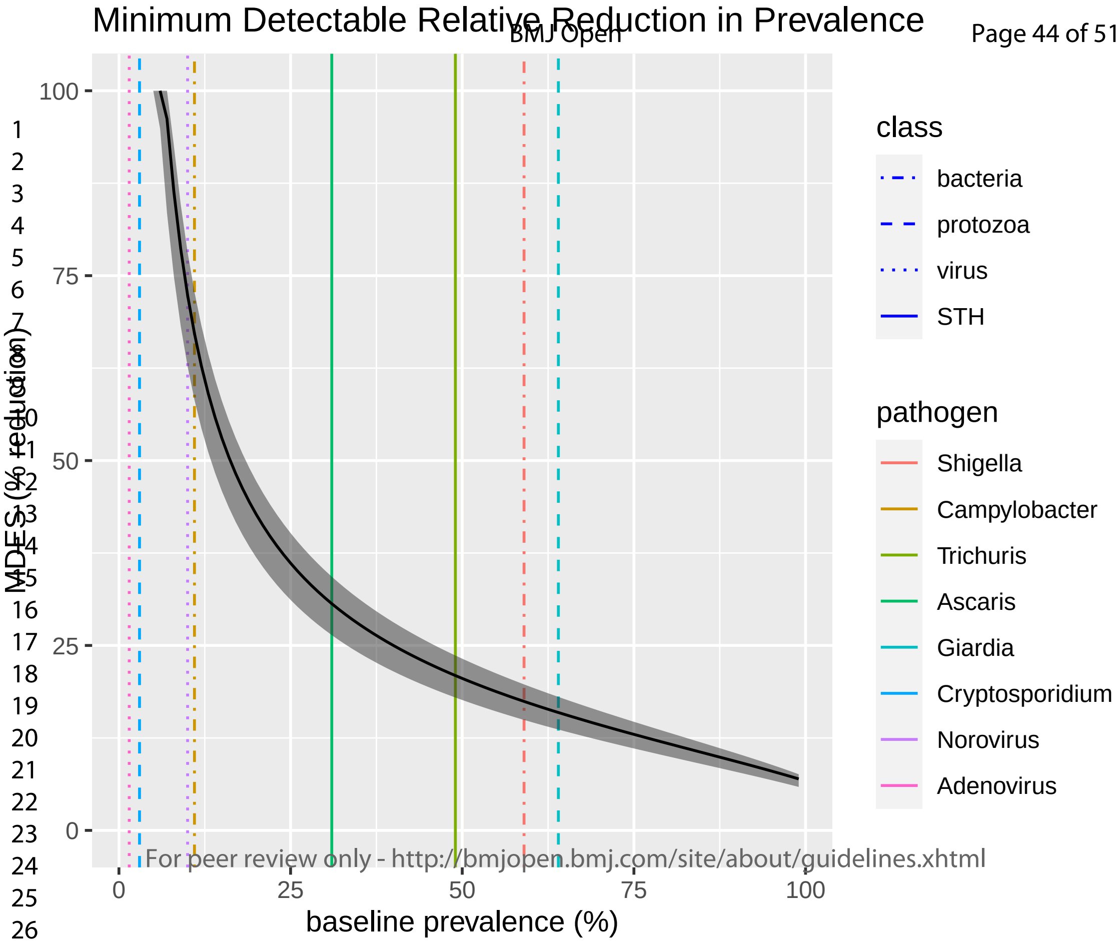

For peer review only - http://bmjopen.bmj.com/site/about/guidelines.xhtml

Table S1. SPIRIT Checklist

		Reporting Item	Page Number
**Administrative information**			
Title	#1	Descriptive title identifying the study design, population, interventions, and, if applicable, trial acronym	1
Trial registration	#2a	Trial identifier and registry name. If not yet registered, name of intended registry	27
Trial registration: data set	#2b	All items from the World Health Organization Trial Registration Data Set	Table S2
Protocol version	#3	Date and version identifier	NA
Funding	#4	Sources and types of financial, material, and other support	28
Roles and responsibilities: contributorship	#5a	Names, affiliations, and roles of protocol contributors	1, 28
Roles and responsibilities: sponsor contact information	#5b	Name and contact information for the trial sponsor	n/a
Roles and responsibilities: sponsor and funder	#5c	Role of study sponsor and funders, if any, in study design; collection, management, analysis, and interpretation of data; writing of the report; and the decision to submit the report for publication, including whether they will have ultimate authority over any of these activities	28
Roles and responsibilities: committees	#5d	Composition, roles, and responsibilities of the coordinating centre, steering committee, endpoint adjudication committee, data management team, and other individuals or groups overseeing the trial, if applicable (see Item 21a for data monitoring committee)	n/a

For peer review only - http://bmjopen.bmj.com/site/about/guidelines.xhtml

		Reporting Item	Page Number
**Introduction**			
Background and rationale	#6a	Description of research question and justification for undertaking the trial, including summary of relevant studies (published and unpublished) examining benefits and harms for each intervention	6-8
Background and rationale: choice of comparators	#6b	Explanation for choice of comparators	11
Objectives	#7	Specific objectives or hypotheses	9
Trial design	#8	Description of trial design including type of trial (eg, parallel group, crossover, factorial, single group), allocation ratio, and framework (eg, superiority, equivalence, non-inferiority, exploratory)	12
**Methods: Participants, interventions, and outcomes**			
Study setting	#9	Description of study settings (eg, community clinic, academic hospital) and list of countries where data will be collected. Reference to where list of study sites can be obtained	10
Eligibility criteria	#10	Inclusion and exclusion criteria for participants. If applicable, eligibility criteria for study centres and individuals who will perform the interventions (eg, surgeons, psychotherapists)	13
Interventions: description	#11a	Interventions for each group with sufficient detail to allow replication, including how and when they will be administered	10-11
Interventions: modifications	#11b	Criteria for discontinuing or modifying allocated interventions for a given trial participant (eg, drug dose change in response to harms, participant request, or improving / worsening disease)	n/a
Interventions: adherance	#11c	Strategies to improve adherence to intervention protocols, and any procedures for monitoring adherence (eg, drug tablet return; laboratory tests)	n/a

For peer review only - http://bmjopen.bmj.com/site/about/guidelines.xhtml

		Reporting Item	Page Number
Interventions: concomitant care	#11d	Relevant concomitant care and interventions that are permitted or prohibited during the trial	21-22
Outcomes	#12	Primary, secondary, and other outcomes, including the specific measurement variable (eg, systolic blood pressure), analysis metric (eg, change from baseline, final value, time to event), method of aggregation (eg, median, proportion), and time point for each outcome. Explanation of the clinical relevance of chosen efficacy and harm outcomes is strongly recommended	16-18
Participant timeline	#13	Time schedule of enrolment, interventions (including any run-ins and washouts), assessments, and visits for participants.	13-14
Sample size	#14	Estimated number of participants needed to achieve study objectives and how it was determined, including clinical and statistical assumptions supporting any sample size calculations	22-23
Recruitment	#15	Strategies for achieving adequate participant enrolment to reach target sample size	12-13
**Methods: Assignment of interventions (for controlled trials)**			
Allocation: sequence generation	#16a	Method of generating the allocation sequence (eg, computer-generated random numbers), and list of any factors for stratification. To reduce predictability of a random sequence, details of any planned restriction (eg, blocking) should be provided in a separate document that is unavailable to those who enrol participants or assign interventions	n/a
Allocation concealment mechanism	#16b	Mechanism of implementing the allocation sequence (eg, central telephone; sequentially numbered, opaque, sealed envelopes), describing any steps to conceal the sequence until interventions are assigned	n/a
Allocation: implementation	#16c	Who will generate the allocation sequence, who will enrol participants, and who will assign participants to interventions	n/a

For peer review only

		Reporting Item	Page Number
Blinding (masking)	#17a	Who will be blinded after assignment to interventions (eg, trial participants, care providers, outcome assessors, data analysts), and how	n/a
Blinding (masking): emergency unblinding	#17b	If blinded, circumstances under which unblinding is permissible, and procedure for revealing a participant's allocated intervention during the trial	n/a
**Methods: Data collection, management, and analysis**			
Data collection plan	#18a	Plans for assessment and collection of outcome, baseline, and other trial data, including any related processes to promote data quality (eg, duplicate measurements, training of assessors) and a description of study instruments (eg, questionnaires, laboratory tests) along with their reliability and validity, if known. Reference to where data collection forms can be found, if not in the protocol	14
Data collection plan: retention	#18b	Plans to promote participant retention and complete follow-up, including list of any outcome data to be collected for participants who discontinue or deviate from intervention protocols	n/a
Data management	#19	Plans for data entry, coding, security, and storage, including any related processes to promote data quality (eg, double data entry; range checks for data values). Reference to where details of data management procedures can be found, if not in the protocol	27
Statistics: outcomes	#20a	Statistical methods for analysing primary and secondary outcomes. Reference to where other details of the statistical analysis plan can be found, if not in the protocol	19-20
Statistics: additional analyses	#20b	Methods for any additional analyses (eg, subgroup and adjusted analyses)	21-22

For peer review only

		Reporting Item	Page Number
Statistics: analysis population and missing data	#20c	Definition of analysis population relating to protocol non-adherence (eg, as randomised analysis), and any statistical methods to handle missing data (eg, multiple imputation)	21
**Methods: Monitoring**			
Data monitoring: formal committee	#21a	Composition of data monitoring committee (DMC); summary of its role and reporting structure; statement of whether it is independent from the sponsor and competing interests; and reference to where further details about its charter can be found, if not in the protocol. Alternatively, an explanation of why a DMC is not needed	n/a
Data monitoring: interim analysis	#21b	Description of any interim analyses and stopping guidelines, including who will have access to these interim results and make the final decision to terminate the trial	n/a
Harms	#22	Plans for collecting, assessing, reporting, and managing solicited and spontaneously reported adverse events and other unintended effects of trial interventions or trial conduct	n/a
Auditing	#23	Frequency and procedures for auditing trial conduct, if any, and whether the process will be independent from investigators and the sponsor	n/a
**Ethics and dissemination**			
Research ethics approval	#24	Plans for seeking research ethics committee / institutional review board (REC / IRB) approval	27
Protocol amendments	#25	Plans for communicating important protocol modifications (eg, changes to eligibility criteria, outcomes, analyses) to relevant parties (eg, investigators, REC / IRBs, trial participants, trial registries, journals, regulators)	27
Consent or assent	#26a	Who will obtain informed consent or assent from potential trial participants or authorised surrogates, and how (see Item 32)	13-14

For peer review only

		Reporting Item	Page Number
Consent or assent: ancillary studies	#26b	Additional consent provisions for collection and use of participant data and biological specimens in ancillary studies, if applicable	27
Confidentiality	#27	How personal information about potential and enrolled participants will be collected, shared, and maintained in order to protect confidentiality before, during, and after the trial	27
Declaration of interests	#28	Financial and other competing interests for principal investigators for the overall trial and each study site	28
Data access	#29	Statement of who will have access to the final trial dataset, and disclosure of contractual agreements that limit such access for investigators	27
Ancillary and post trial care	#30	Provisions, if any, for ancillary and post-trial care, and for compensation to those who suffer harm from trial participation	n/a
Dissemination policy: trial results	#31a	Plans for investigators and sponsor to communicate trial results to participants, healthcare professionals, the public, and other relevant groups (eg, via publication, reporting in results databases, or other data sharing arrangements), including any publication restrictions	27
Dissemination policy: authorship	#31b	Authorship eligibility guidelines and any intended use of professional writers	n/a
Dissemination policy: reproducible research	#31c	Plans, if any, for granting public access to the full protocol, participant-level dataset, and statistical code	27
**Appendices**			
Informed consent materials	#32	Model consent form and other related documentation given to participants and authorised surrogates	n/a
Biological specimens	#33	Plans for collection, laboratory evaluation, and storage of biological specimens for genetic or molecular analysis in the current trial and for future use in ancillary studies, if applicable	n/a

For peer review only

Table S2. World Health Organization Trial Registration Data Set

Data category	Information
Primary registry and trial identifying number	ISRCTN 86084138<br>https://doi.org/10.1186/ISRCTN86084138
Date of registration in primary registry	16 March, 2022
Secondary identifying numbers	n/a
Source(s) of monetary or material support	Bill & Melinda Gates Foundation; National Institute of Environmental Health Sciences
Primary sponsor	n/a
Secondary sponsor(s)	n/a
Contact for public queries	Joe Brown, PhD PE; joebrown@unc.edu
Contact for scientific queries	Joe Brown, PhD PE<br>Department of Environmental Sciences and Engineering<br>University of North Carolina at Chapel Hill
Public title	Health impacts on children born after an urban sanitation intervention in low-income neighborhoods in Mozambique: the Maputo Sanitation (MapSan) trial after five years
Scientific title	Long-term impact of an urban sanitation intervention on child health in low-income neighborhoods of Maputo city, Mozambique: a cross-sectional follow-up five years post-intervention in the Maputo Sanitation (MapSan) trial
Countries of recruitment	Mozambique
Health condition(s) or problem(s) studied	Shedding of enteric pathogens in stool and associated sequelae in children under 5 years of age
Intervention(s)	Intervention: existing onsite (non-sewered) pour-flush toilet to septic tank and soak pit for liquid effluent
	Control: comparable household clusters with unhygienic shared sanitation facilities in poor condition
Key inclusion and exclusion criteria	Ages eligible for study: 29 days – 60 months<br>Sexes eligible for study: both<br>Accepts healthy volunteers: yes
	Inclusion criteria: born into and residing in a compound enrolled in the five-year follow-up study; continuously resided in the study compound for the preceding 6 months, or since birth if under 6 months of age; parent or guardian who is able to understand and complete the written informed consent process and allow their child to participate
	Exclusion criteria: any caregiver-indicated medical condition or disability that precludes participation in the study
Study type	Observational, cross-sectional
	Allocation: non-randomized, unblinded
	Primary purpose: prevention

For peer review only - http://bmjopen.bmj.com/site/about/guidelines.xhtml

Data category	Information
	Trial setting: home
Date of first enrolment	28 March 2022
Target sample size	1100
Recruitment status	Recruiting
Primary outcome(s)	Prevalence of 22 bacterial, protozoan, and soil transmitted helminth (STH) enteric pathogens in child stool
Key secondary outcomes	Individual pathogen detection prevalence and gene copy density of 27 enteric pathogens (including viruses); mean height-for-age (HAZ), weight-for-age (WAZ), and weight-for-height (WHZ) z-scores; prevalence of stunting, underweight, and wasting; and the 7-day period-prevalence of caregiver-reported diarrhea.

For peer review only
